# Understanding lncRNAs: key regulators of myogenesis and lipogenesis in farm animals

**DOI:** 10.3389/fvets.2025.1540613

**Published:** 2025-02-14

**Authors:** Wenjing Liu, Mengjie Chen, Yining Liu, Xinxin Li, Hui Li, Jian Wang

**Affiliations:** ^1^Guangxi Key Laboratory of Animal Breeding, Disease Control and Prevention, College of Animal Science and Technology, Guangxi University, Nanning, China; ^2^Institute of Scientific Research, Guangxi University, Nanning, China

**Keywords:** lncRNAs, biogenesis, farm animals, myogenesis, lipogenesis

## Abstract

Long non-coding RNAs (lncRNAs) are RNA molecules exceeding 200 nucleotides in length. Recent studies have demonstrated their involvement in regulating gene expression and various biological processes. Among these, myogenesis and lipogenesis are particularly important because of their direct effects on muscle development and fat deposition in farm animals. These processes are crucial for determining meat quality, growth rates, and overall economic value in animal husbandry. Although the specific mechanisms through which lncRNAs influence these pathways are still under investigation, further research into their roles in muscle and fat development is crucial for optimizing farm animal breeding strategies. Here, we review the characteristics of lncRNAs, including their biogenesis, localization, and structures, with a particular focus on their association with myogenesis and adipogenesis. This review seeks to establish a theoretical foundation for enhancing farm animal production. In particular, focusing on lncRNAs may reveal how these molecules can enhance the economic traits of farm animals, thereby contributing to the optimization of farm animal breeding processes.

## 1 Introduction

Farm animals are important as they can supply the basic nutritional needs of humankind, including meat, eggs, and milk. Additionally, animal products such as gelatin are widely used as food additives in confectionery, and farm animal waste serves as an excellent fertilizer. Optimizing farm animal production is crucial to meet the ever-growing demand for animal products. A key strategy would be to improve the amount and quality of farm animal products by understanding the molecular mechanisms of the key biological processes that govern animal reproduction and well-being. The mechanisms that regulate skeletal muscle growth, development, and fat deposition are critical determinants of meat yield and quality.

Numerous studies have attempted to elucidate the molecular mechanisms underlying various animal traits, especially skeletal muscle and adipose tissue development. For instance, myogenesis regulators such as *MyoD*, *Myf5*, *Myogenin* (*MyoG*), and *MRF4* are crucial for skeletal muscle development, satellite cell activation, and regenerative myogenesis (1). In addition, genetic variations in fatty acid synthesis and deposition between different cattle breeds have been observed to influence beef marbling (2). Recent studies have shown that traits in farm animals, such as muscle (3–6) and fat development (7–11), are influenced not only by coding genes but also by the regulatory roles of long non-coding RNAs (lncRNAs).

LncRNAs are a type of non-coding RNA (ncRNA) molecule exceeding 200 nucleotides (nts) in length and are known to significantly influence various traits in farm animals. For example, lncRNA muscle growth promoting factor (lncMGPF) is a conserved lncRNA found in pigs, which promotes muscle growth and regeneration by enhancing HuR-mediated mRNA stability of myogenic regulators and acting as a molecular sponge for miR-135a-5p (12). LncCCPG1 in bovine adipose cells can alleviate the inhibition of lncSLC30A9 expression by miR-93 through miR-93 adsorption LncSLC30A9 inhibits cell proliferation by downregulating AKT expression and promotes bovine adipocyte differentiation through the recruitment of FOS proteins to the peroxisome proliferation-activated receptor gamma (PPARγ) promoter (8). A highly specific sheep enhanced muscularity Transcript lncRNA (lnc-SEMT) has been identified in sheep skeletal muscle tissue. Lnc-SEMT acts as a molecular sponge by antagonizing miR-125b to control IGF2 protein abundance and promote sheep myoblast differentiation *in vitro* (13). In chicken skeletal muscle, a lncRNA named myosin, heavy chain 1G (MYH1G)-antisense transcript (MYH1G-AS) has been identified. It promotes the transcription of *SMAD4* by reducing the interaction between FGF18 and SMARCA5. This action activates the *SMAD4*-dependent pathway, thereby enhancing the proliferation of myoblasts (14). This review will examine recent progress in understanding the role of lncRNAs in skeletal muscle development and fat deposition in different farm animal species. It will also delve into their regulatory mechanisms at the epigenetic, transcriptional, and post-transcriptional levels.

## 2 Biogenesis of lncRNAs

**Figure 1:**
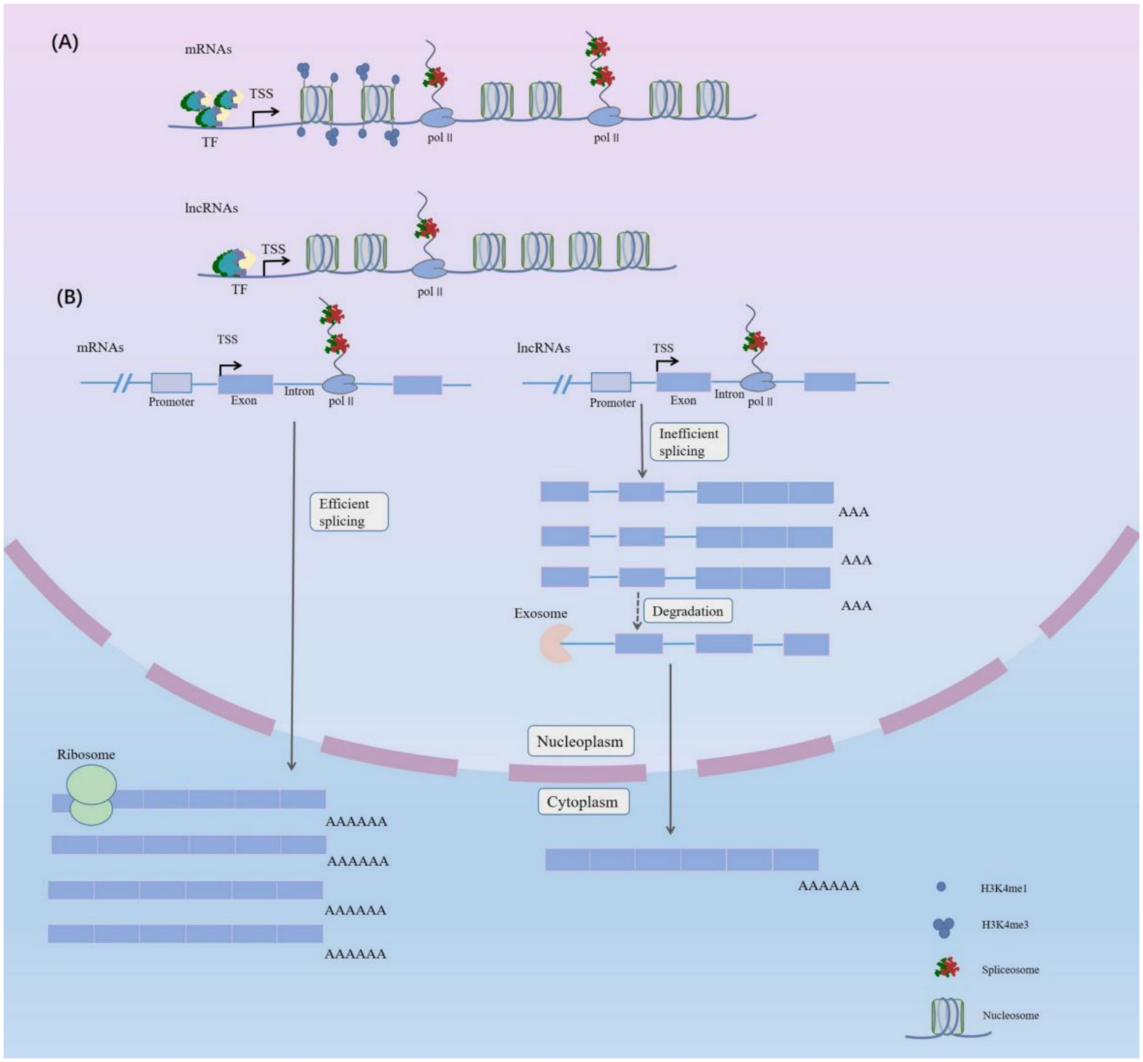
Distinguishing the Characteristics of Long Noncoding RNAs and mRNAs. **(A)** Compared with mRNAs, lncRNAs have fewer transcription factors (TFs) bound to their promoters. In addition, the splicing efficiency of lncRNAs is lower than that of mRNAs. mRNAs are associated with H3K4me1 (associated with enhancers) and H3K4me3 modifications (associated with promoters). In addition, lncRNAs are characterized by enrichment of H3K9me3 modifications at the promoter site, which correlates with tissue specificity. RNA Pol II, RNA polymerase II; TSS, transcription start site. **(B)** Unlike mRNAs, many lncRNAs transcribed by RNA polymerase II (Pol II) are processed inefficiently and most remain in the nucleus. Only a small proportion which are similar to mRNAs enters the cytoplasm, and some lncRNAs which are located in the nuclei are degraded by exosomes. While mRNAs are more abundant in the cytoplasm and these are invariably associated with ribosomes.

There are several ways to form lncRNAs in living cells (15): (1): Protein-coding gene open reading frames (ORFs) can be cut and mutated to produce lncRNAs (15); (2) Chromatin rearrangement can cause two distant untranscribed fragments to concatenate, generating multi-exon lncRNAs (15); (3) Retro-transposition can produce lncRNAs (15); (4) The same sequence can be duplicated resulting in the production of lncRNAs with adjacent repeating sequences (15); (5) Insertion of transposable elements can produce functional lncRNAs (15); (6) Enhancer transcription can produce enhancer-associated lncRNAs (elncRNAs) (16); (7) The upstream region of a promoter can be transcribed in order to generate short-lived lncRNAs (17); (8) Excised intron-derived small nucleolar RNA (snoRNA)-ended can give rise to some lncRNAs (18, 19). LncRNAs, similar to protein-coding genes, have conserved core promoter sequences. However, because there are fewer overlapping motifs bound by TFs in lncRNA promoters (Figure 1A), the expression levels of lncRNAs are generally lower than those of protein-coding genes (20). The DNA core promoter initiates transcription, which results in the production of mRNAs and lncRNAs. The pre-mRNAs are transcribed by Pol II and must undergo 5′ capping, splicing and 3′ cleavage and polyadenylation. Therefore, the lncRNAs produced are frequently cleaved and prematurely terminated during co-transcription. Although the splicing mechanisms of lncRNAs resemble those of protein-coding genes, the splicing efficiency in lncRNAs is typically reduced (Figure 1B).

## 3 The roles of lncRNAs in the regulation of gene expression

**Figure 2:**
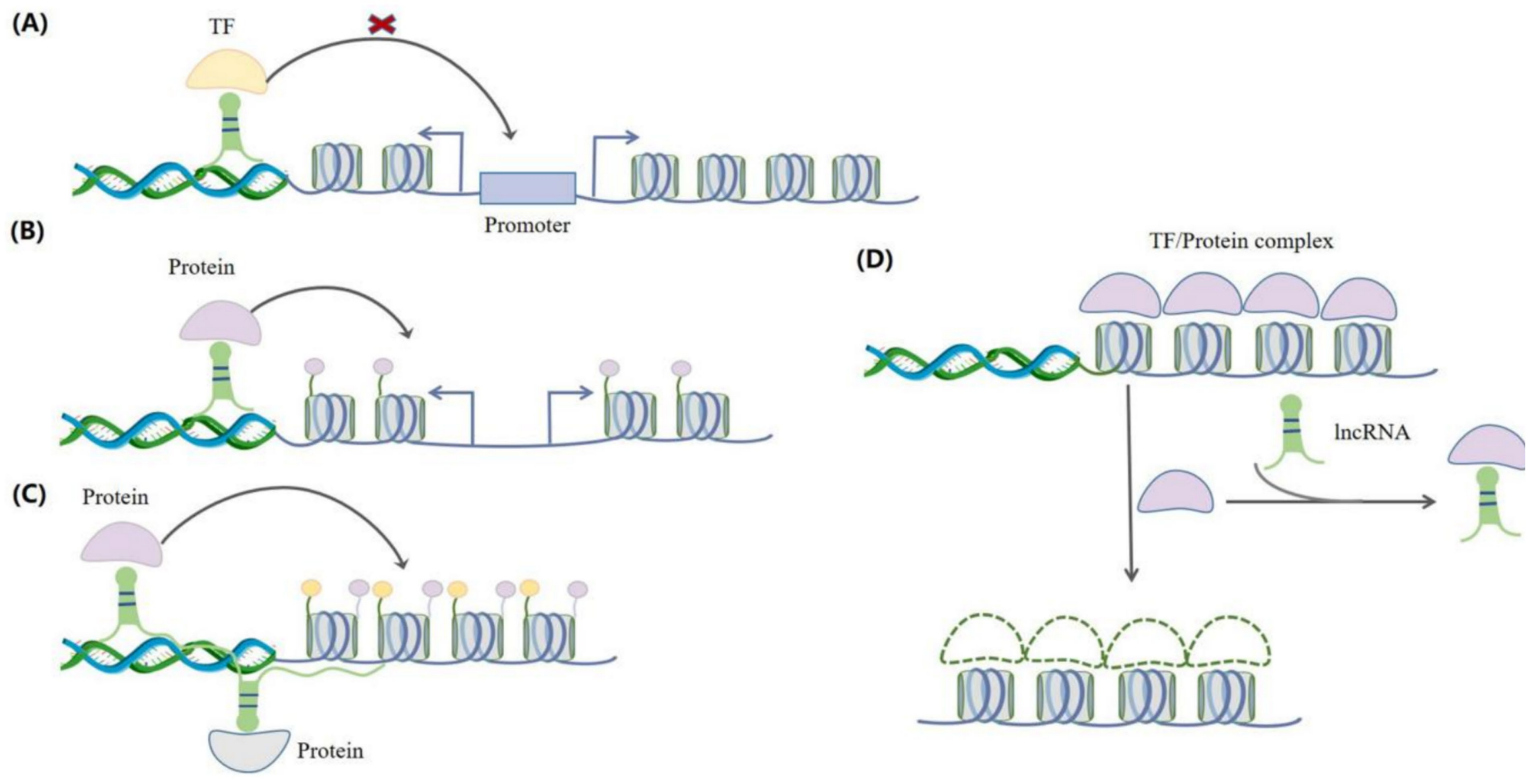
Interactions between lncRNAs and regulatory factors. **(A)** As guide molecules, lncRNAs can bind to transcription factors to prevent them from attaching to the promoters of target genes, thereby regulating gene expression. **(B)** As guide molecules, lncRNAs can recruit epigenetic regulators (such as proteins) to chromatin and regulate the expression of target genes. **(C)** As scaffold molecules, lncRNAs can recruit different protein complexes to regulate target gene expression. **(D)** LncRNAs can also act as decoys for transcription factors and proteins, preventing them from binding to chromatin, thereby regulating gene expression.

LncRNA can function through multiple ways to regulate gene expression. They can interact with proteins, RNAs, and DNAs, acting as guides (8), scaffolds (21), and bait (22) molecules in order to regulate transcription (Figure 2). This section investigates the functions of lncRNAs in the contexts of epigenetics, transcription, and post-transcription.

### 3.1 The roles of lncRNAs in epigenetic level regulation

In eukaryotes, chromatin is highly folded and compressed, which reduces its ability to bind to TFs, promoters, and enhancers. Dynamic alterations of the chromatin structure can promote its accessibility. One of the key determinants of transcriptional activity is the state of the chromatin, and lncRNAs can regulate this parameter in several ways to mediate gene transcription and silencing. For instance, lncRNAs interact with proteins, histone-modifying enzymes, and chromatin-modifying complexes (23) and are involved in chromatin epigenetic regulation, such as chromatin remodeling (24), gene imprinting (25), and dosage compensation (26).

**Figure 3:**
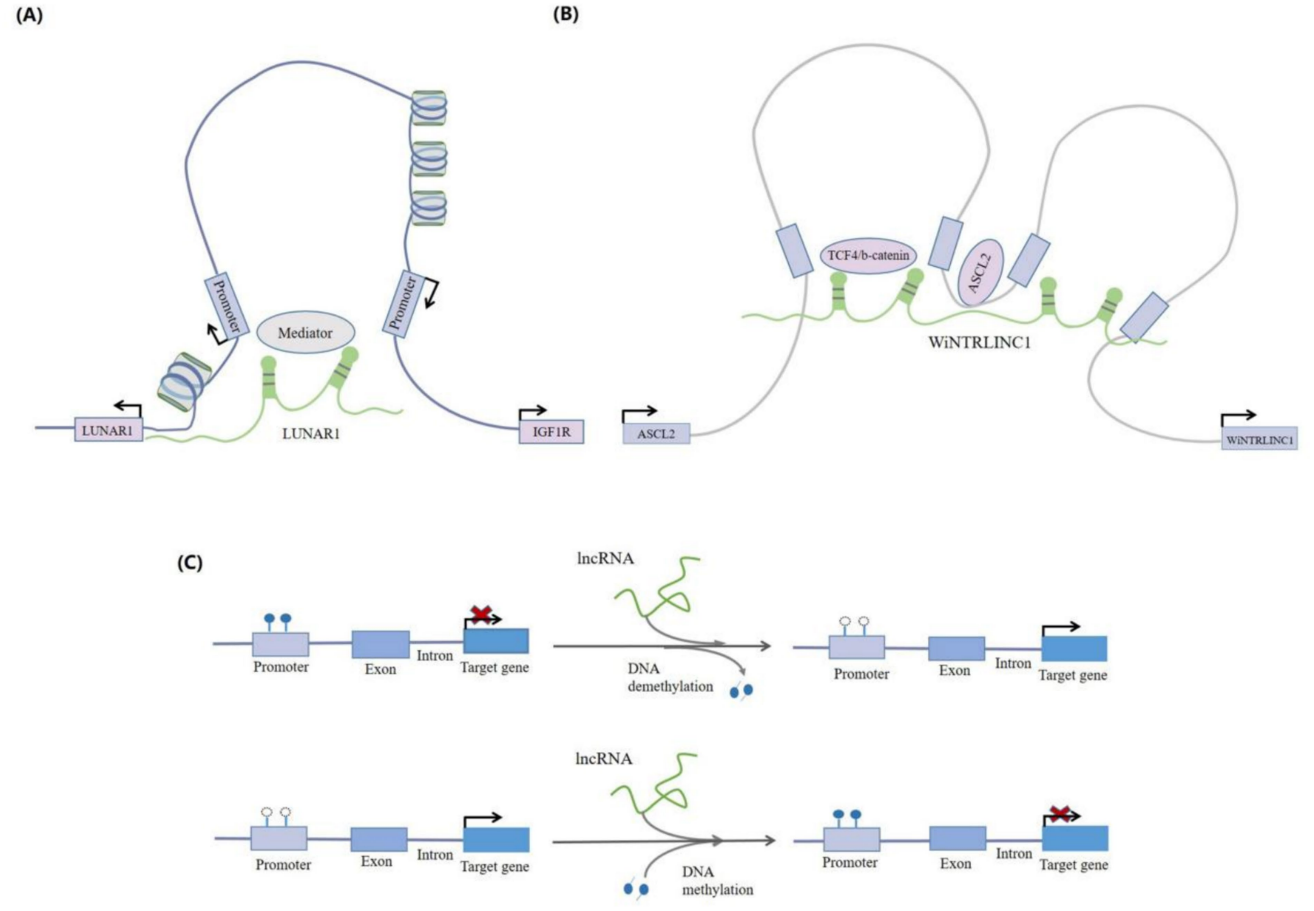
LncRNA-mediated chromatin regulation. **(A)** LncRNAs can interact with chromatin modification complexes and recruit them to target gene promoters in order to activate or repress the transcription of target genes. For example, the lncRNALUNAR1 can promote and interact with chromosome looping, and activate the transcription of IGF1R by recruiting the chromatin modification complex mediator to the promoter region of the IGF1R gene. **(B)** The lncRNA, WiNTRLINC1K, can bind to the TCF4/b-catenin complex and mediate the formation of chromatin loops between the promoter of WiNTRLINC1 and the regulatory domain of transcription factor, ASCL2. In turn, ASCL2 can bind to the WiNTRLINC1 promoter and positively regulate WiNTRLINC1 transcription. **(C)** LncRNAs can bi-directionally regulate DNA methylation levels. They can, not only promote DNA methylation in promoter regions (such as lncRNA Xist), but also play a role in DNA demethylation.

LncRNAs can organize chromatin domains to coordinate gene activation. LncRNAs can regulate gene expression by recruiting chromatin-modifying complexes, such as PRC2, G9a, and hnRNPK, to specific gene loci for chromatin remodeling. The HOX Transcript Antisense Intergenic RNA (lncRNA HOTAIR) recruits PRC2, leading to histone H3 lysine-27 trimethylation (H3K27me3) and transcriptional silencing of a ~ 40-kb region at the *HOXD* locus in various human fibroblasts cultured *in vitro* (24). Chromatin looping involves alterations in the three-dimensional structure of chromatin. LncRNAs promote the construction of chromatin loops by recruiting specific chromatin modification complexes (27, 28), which can alter the expression of adjacent genes (Figures 3A,B). LncRNA HOTTIP attracts the MLL1 protein’s histone lysine methyltransferase complex by directly binding to the WDR5 protein. This targets *MLL1* to the *HOXA* site by chromatin looping, which then induces histone H3 lysine 4 trimethylation (H3K4me3) and promotes *HOXA* gene transcription (27).

DNA methylation silencing is a common form of epigenetic regulation that suppresses gene transcription. LncRNAs often influence gene expression by modulating the methylation status of CpG islands within promoter regions. DNA methylation silencing is a common epigenetic regulatory mechanism that inhibits gene transcription. LncRNAs regulate DNA methylation in a bidirectional manner, promoting both DNA methylation and demethylation (Figure 3C). For instance, LncRNA CRNDE enhances *NDRG2* expression through DNA methylation in B lymphocytes, thereby inhibiting cell proliferation and promoting apoptosis (29).

### 3.2 The role of lncRNAs in transcriptional regulation

Gene transcription is a rigorous and complex biological process, and lncRNAs can regulate it through various mechanisms. The multidimensional mechanisms by which lncRNAs participate in gene regulation suggest that we can only learn more about how lncRNAs are used for gene regulation by elucidating the RNA sequences and structural elements that make the lncRNAs functional.

**Figure 4:**
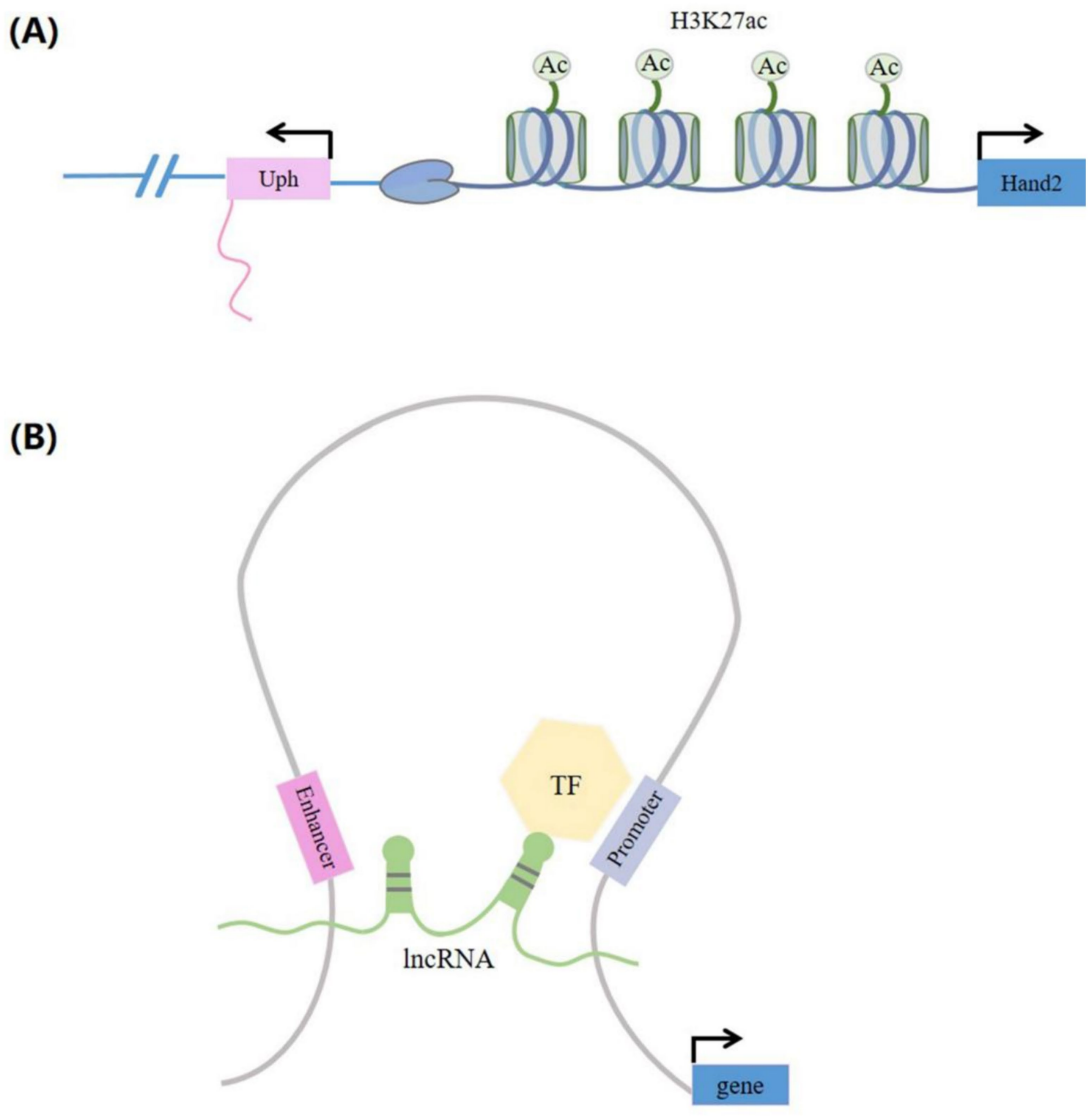
LncRNAs produced through enhancer-mediated gene activation. **(A)** When the lncRNAs were transcribed, the H3K27ac active enhancer tag in the Uph locus is guaranteed to activate transcription of the proximal Hand2 gene. **(B)** LncRNAs have the function of activating gene expression. LncRNAs can promote enhancer cyclization and are able to recruit chromatin-activated complexes (such as transcription factors) to the promoter of the protein coding genes. These are capable of regulating the transcription and expression of target genes. TF, transcription factors.

LncRNAs can modulate gene transcription by either recruiting TFs to target the gene promoter regions or by interfering with Pol II at the targeted loci. When lncRNAs are transcribed, they can interfere with TF binding to the promoter, thus inhibiting gene transcription. A case of this kind of transcriptional interference effect is when the lncRNA SRG1, upstream of the yeast gene *SER3*, straddles the *SER3* promoter sequence during transcriptional elongation, sequestering Pol II from binding to the *SER3*. This results in the repression of *SER3* transcription (30). LncRNAs can also influence gene transcription by functioning as co-transcriptional factors (co-TFs). For instance, the lncRNA Evf2 interacts with the TF DLX2, forming a complex that enhances the transcriptional activity of the *Dlx-5/6* enhancer, thereby regulating the expression of *Dlx5* and *Dlx6* (31). LncRNAs can also influence gene transcription by hybridizing with DNA to form triple helices, recruiting transcriptional cofactors to target gene promoter regions. For example, the lncRNA Khps1 generates a DNA–RNA triplet with the upstream region of the *SPHK1* enhancer, which in turn promotes the recruitment of the histone acetyltransferase p300/CBP to the *SPHK1* promoter. This process activates *SPHK1*-eRNA transcription and enhances *SPHK1* expression (32). Numerous studies have demonstrated that DNA elements like enhancers (33) and promoters (34) located at lncRNA sites, rather than lncRNA transcripts, play a regulatory role in gene transcription (Figure 4A). For instance, a DNase hypersensitive site on the Lockd locus promoter interacts with multiple TFs and regulates *Cdkn1b* gene expression by binding to the *Cdkn1b* promoter. A study demonstrated a significant reduction in *Cdkn1b* expression following the deletion of the 25 kb Lockd locus. However, the reduction of Lockd lncRNA transcripts did not affect the expression of *Cdkn1b* (34). In addition, lncRNAs can promote enhancer circularization and regulate gene transcription and expression by recruiting chromatin activation complexes to target gene promoters (Figure 4B). For example, the Colon Cancer Associated Transcript 1, the Long isoform (lncRNA CCAT1L), situated within a super-enhancer region, regulates *MYC* transcription by facilitating the long-range interaction between the *MYC* promoter and its enhancer (28).

### 3.3 The roles of lncRNAs in post-transcriptional regulation

LncRNAs are crucial in the regulation of RNA splicing. Specifically, metastasis-associated lung adenocarcinoma transcript 1 (lncRNA-MALAT1) influences the alternative splicing of precursor mRNA by affecting the activity of serine/arginine-rich splicing factors (35). Additionally, lncRNAs can be cleaved and processed into smaller non-coding RNAs, such as miRNAs and piwi-interacting RNAs (piRNAs), which are involved in regulating gene expression after transcription.

**Figure 5:**
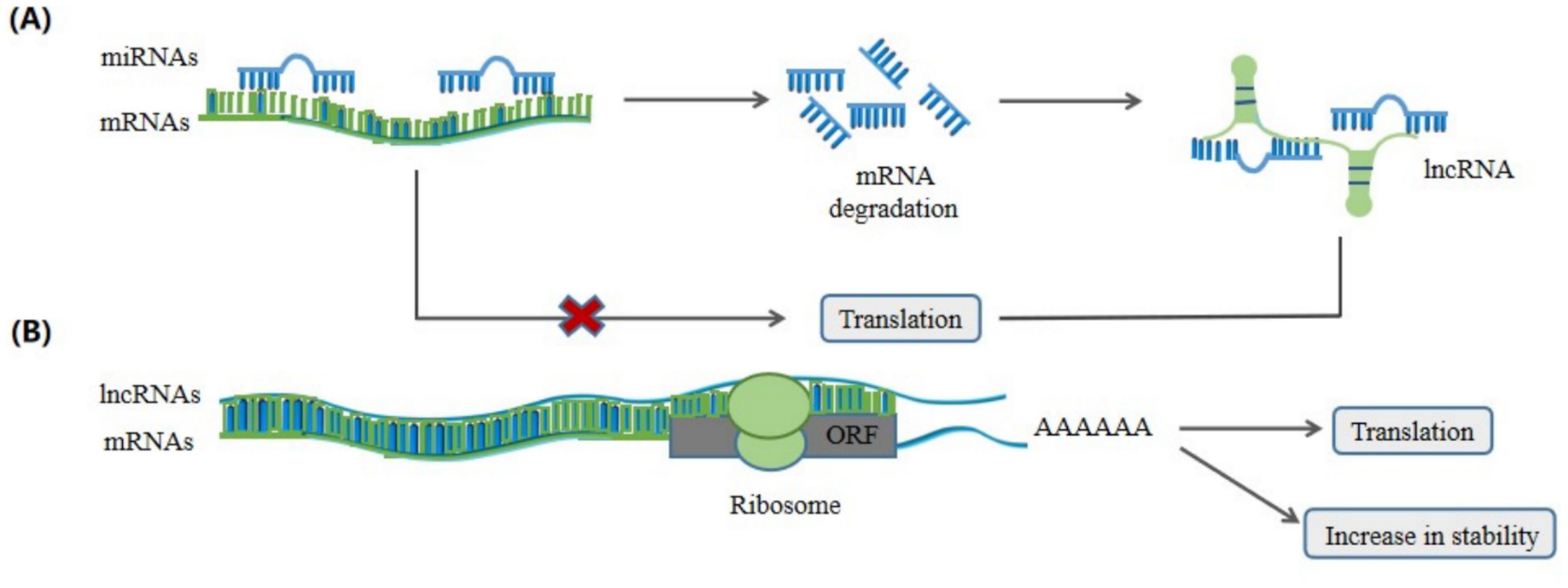
LncRNAs regulate mRNA degradation, stability and translation. **(A)** MiRNAs bind to mRNAs to regulate the degradation and translation of mRNAs, while lncRNAs can prevent miRNAs from degrading the target gene mRNAs and regulate mRNAs translation by absorbing to microRNAs (miRNAs). **(B)** Cytoplasmic lncRNAs can bind to mRNAs to form double-stranded RNAs in order to increase the stability and regulate the translation of the mRNAs.

Furthermore, LncRNAs can regulate RNA activity by interacting with proteins (36) and RNAs (37). For example, LncMyoD directly binds to IGF2-mRNA-binding protein 2 (IMP2) and negatively regulates IMP2-mediated translation of proliferation genes such as *N-Ras* and *c-Myc* (36). LncRNAs can regulate mRNA stability by absorbing miRNAs (Figure 5A). For example, intramuscular fat deposition-associated long noncoding RNA 1 (lncRNA IMFlnc1) binds to miR-199a, preventing it from degrading the target gene, caveolin-1 (*CAV-1*), and promoting adipogenesis (37). LncRNAs can influence mRNA stability and translation by binding to mRNAs through base pairing interactions (Figure 5B). For example, PU.1 AS lncRNA can form a sense-antisense RNA duplex with *PU.1* mRNA, thereby inhibiting the translation of porcine *PU.1* mRNAs (38).

Moreover, extensive research has shown that lncRNAs can encode small functional peptides. Most lncRNAs are classified as ncRNAs because they typically contain only small open reading frames (smORFs), ORFs with 100 codons or less, potentially able to be translated into proteins shorter than 100 aa before post-translational modifications (such as proteolytic cleavage) (39). However, recent research has indicated that lncRNAs can encode small peptides, which have been found to be critical for muscle growth (40) and relaxation (41, 42). By interacting with lysosomal v-type ATPase, the short peptide, SPAR, encoded by LINC00961, can limit muscle regeneration by selectively reducing the ability of mTORC1 to recognize amino acid stimulation (40).

## 4 Research progress in farm animals

Pigs, cattle, sheep and poultry are four key farm animals that provide abundant animal products for population worldwide, including meat, eggs and milk. LncRNAs have been demonstrated to influence economic traits in farm animals, though research on their role in these animals remains in the early stages. In this section, we review the recent studies that explore the functions and mechanisms of lncRNAs on myogenesis, lipogenesis in the aforementioned four farm animals.

### 4.1 The roles of lncRNAs in myogenesis of farm animals

Skeletal muscle is the main component of animal bodies, and its formation and structure directly influence meat yield and quality. Recent decades of research have underscored the vital role of lncRNAs in the development of skeletal muscle (Table 1).

**Table 1:** LncRNA-mediated regulation of muscle tissues.

LncRNAs	Cell localization	Role in myogenesis	Partner	Species	Reference
Dum	Nucleus/Cytoplasm	DNA methyltransferase inhibits Dppa2 transcription through its neighboring gene Dppa2, and promotes myoblast differentiation and muscle regeneration.	DUMTs	*Mus musculus*	(124)
LncYY1	Nucleus	By recruiting PRC2 to myogenic genes, thereby inhibiting myoblast differentiation. Remove YY1/PRC2 to stimulate myogenic activity genes to promote the differentiation of myoblasts.	YY1	*Mus musculus*	(125)
Gtl2 (Meg3)	Nucleus	As a cofactor of PRC2, it can promote the combination of PRC2 and Dlk1 gene and inhibit its expression to regulate the development of skeletal muscle.	PRC2	*Mus musculus*	(126)
Malat1	Nucleus/Cytoplasm	Suv39h1 was recruited to the MyoD binding site to inhibit myoblast differentiation.	MiR-133/miR-181a/Suv39h1	*Mus musculus*	(19)
RAM	Nucleus/Cytoplasm	Directly combines with MyoD to promote muscle cell differentiation.	MyoD	*Mus musculus*	(127)
SRA	Nucleus	As a molecular scaffold for the co-activation complex, RNA helicase coregulators P68, P72 and MyoD.	p68/p72/MyoD	*Mus musculus*	(128)
MUNC (^DRR^RNA)	Nucleus	Muscle differentiation can be regulated by regulating the transcriptional expression of MyoD promoters combined with DRR.	MyoD	*Homo sapiens*	(129)
^CE^RNA	Nucleus	Regulate the chromatin remodeling of the MyoD promoter and the recruitment of Pol II, regulate the expression level of MyoD and then regulate muscle development.	MyoD	*Mus musculus*	(130)
Six1	Nucleus/Cytoplasm	It regulates its neighboring gene Six1 in cis to promote the expression of genes related to muscle growth.	Six1	*Gallus gallus*	(86)
LncMD1	Cytoplasm	By competitively binding miR-133 and miR-135 to regulate the expression of muscle-specific genes MAML1 and MEF2C, thereby regulating the differentiation of muscle cells.	MiR-135/miR-133	*Homo sapiens*、 *Mus musculus*	(131)
H19	Nucleus/Cytoplasm	Regulates muscle differentiation by acting as a sponge for let-7.	Let-7	*Homo sapiens*、 *Mus musculus*	(132)
Lnc-mg	Nucleus/Cytoplasm	As a molecular sponge of miR-125b, it controls the protein level of IGF2, thereby affecting the myogenic differentiation of mice.	MiR-125b	*Mus musculus*	(133)
LncMD	Nucleus/Cytoplasm	By absorbing miR-125b to increase the expression level of insulin like growth factor 2 (IGF2) to promote the differentiation of bovine myoblasts.	MiR-125b	*Bos taurus*	(134)
Yam1	Nucleus/Cytoplasm	By activating the expression of miR-715 to inhibit the differentiation of myoblasts, miR-715 targets Wnt7b, which promotes skeletal muscle differentiation, to promote muscle development.	MiR-715	*Mus musculus*	(135)
LncMyoD	Nucleus/Cytoplasm	By competing with IGF2 mRNA to bind IMP2 protein, thereby blocking the cell cycle and promoting myoblast differentiation.	IMPs	*Mus musculus*	(36)
1/2-sbsRNAs	Cytoplasm	It controls myogenesis by base pairing with the 3’-UTR of ARF mRNA and triggering SMD.	SINE-contai ning mRNA 3’ UTRs	*Mus musculus*	(136)
Sirt1 AS	Nucleus/Cytoplasm	Stabilizes Sirt1 mRNA through competitive binding with miR-34a, thereby promoting the proliferation of myoblasts.	Sirt1 mRNA	*Mus musculus*	(137)
MLN	SR/ER membrane	It interacts with SERCA to control muscle relaxation by regulating the uptake of calcium ions by SR.	SERCA	*Mus musculus*	(41)
DWORF	SR membrane	Improves SERCA activity by replacing SERCA inhibitors, enhance SR Ca^2+^ uptake and myocardial cell contractility.	SERCA	*Mus musculus*	(42)
DBE-T	Nucleus	DBE-T binds to the TrxG protein Ash1L and recruits it to the FSHD site, leading to cis-disinhibition of nearby genes.	TrxG protein Ash1L	*Homo sapiens*	(138)
MGPF	Nucleus/Cytoplasm	Promote muscle growth and regeneration by acting as a miR-135a-5p molecular sponge.	MiR-135a-5p	*Sus scrofa*	(12)
IRS1	Nucleus/Cytoplasm	Promotes IRS1 gene expression by absorbing the miR-15 family, activate the IGF1-PI3K/AKT signaling pathway, and promote the proliferation and differentiation of chicken myoblasts.	MiR-15	*Gallus gallus*	(85)
IGF2 AS	Nucleus/Cytoplasm	Directly binds with ILF3 protein to affect the expression of genes related to muscle proliferation and differentiation to regulate cattle muscle production.	ILF3	*Bos taurus*	(3)
SYISL	Nucleus/Cytoplasm	The recruitment of PRC2 protein leads to the occurrence of H3K27me3 in the promoter region of the target gene, thereby promoting the proliferation and fusion of myoblasts, while inhibiting myogenic differentiation.	PRC2	*Mus musculus*	(139)
MDNCR	Nucleus/Cytoplasm	Combining with miR-133a to inhibit the expression of GosB promotes myoblast differentiation and inhibits cell proliferation.	MiR-133a	*Bos taurus*	(67)
Lnc-smad7	Nucleus/Cytoplasm	Promotes myoblast differentiation and promotes skeletal muscle regeneration by acting as a competitive endogenous RNA of miRNA-125b.	MiRNA-125b	*Mus musculus*	(140)
Lnc23	Nucleus	Reduces the inhibitory effect of PFN1 on RhoA and Rac1 by binding to PFN1, thereby promoting myogenesis in bovine skeletal muscle satellite cells.	PFN1	*Bos taurus*	(115)

#### 4.1.1 The roles of lncRNAs in myogenesis of pigs

NONCODE is a comprehensive knowledge database focusing on ncRNA genes. Among farm animals, this database^1^ contains the highest number of lncRNA transcripts in pigs, totaling 29,585. In recent years, researchers have studied the expression of several lncRNAs in porcine muscle tissues (11, 43–47). They have examined the differential expression of lncRNAs between muscle and other tissues (48), in cloned and normal breeding piglets (49), and in gene-edited and normal pigs (50). They have identified numerous lncRNAs linked to the development of porcine muscle in research on pig breeds with varying growth rates (43–46). Specifically, 1,407 differentially expressed lncRNAs (DELs) were found in the skeletal muscles of pigs with distinct muscle growth rates throughout their lifespans (43). Both RNA-seq and miRNA-seq techniques were used to analyze the expression of ncRNAs in the *longissimus dorsi* muscles (*LDMs*) of pigs (46). By predicting quantitative trait loci (QTL) for the DELs, it was observed that most of them were associated with muscle development (45).

The quality of pork, including color, water retention and tenderness, has been a concern for decades. Breed is a significant factor that can affect meat quality in various ways, such as total fiber count, fiber cross-sectional area (CSA), and the composition of fiber types in a specific muscle within the species. The differential expression of lncRNAs among different pig breeds may be crucial in accounting for differences in growth rates and meat quality among these breeds (44). In the study of lncRNAs in the longest muscle of pigs from the same breed at different life stages (11, 47), QTL mapping analysis of the DELs identified several loci associated with growth and meat quality traits (47). Since vertebrate skeletal muscle consists mainly of muscle fibers, the quality of fresh meat is closely linked to these fibers’ characteristics. Morphological features, including total fiber number (TNF) and (CSA), are key determinants of muscle mass and meat quality (51). In addition, the quality of fresh meat was closely correlated with the fiber type component (FTC) within muscle tissue (51). One study identified 92 differentially expressed lncRNAs in the muscles of fast-twitch *Biceps femoris* (Bf) and slow-twitch *Soleus* (Sol). The study found that lncRNA MSTRG.42019 is linked to fiber types in porcine skeletal muscle and associated with meat quality traits (52).

Meat production can be directly increased by increasing the cross-sectional area of muscle fibers (53, 54). Biological pathways that effectively increase meat yield include promoting cell proliferation, differentiation, and muscle cell hypertrophy, leading to increased muscle fiber cross-sectional area. Research has shown that lncRNAs influence the proliferation and differentiation of porcine muscle cells via diverse mechanisms, thereby regulating muscle development at the epigenetic level. LncMREF, a conserved lncRNA found in both humans and pigs, promotes myogenic differentiation and muscle regeneration through its interaction with Smarca5/p300 complexes. This interaction leads to the upregulation of key myogenesis regulators, including MyoD (55). Certain lncRNAs, such as MSTRG.59589, can impact biological processes by modulating the expression of adjacent genes. Specifically, lncRNA MSTRG.59589 increases *PALLD* expression and promotes the differentiation of porcine skeletal muscle satellite cells (PSCs) (5). As members of the ceRNA (competitive endogenous RNA) family, lncRNAs play a critical regulatory role in the growth of porcine muscle. For example, lncRNA maternally expressed gene 3 (lncRNA MEG3) can competitively bind to miR-423-5p, thereby upregulating serum response factor (*SRF*) expression and facilitating the differentiation of porcine skeletal muscle (6). Some lncRNAs are known to regulate muscle atrophy in pigs. Synaptopodin-2 (SYNPO2) intron sense-overlapping lncRNA (SYISL), a conserved lncRNA, has been shown to regulate myogenesis across mice, humans, and pigs. Additionally, *SYISL* can promote muscle atrophy by interacting with miR-23a-3p/miR-205-5p, and miR-103-3p (56). Interestingly, certain lncRNAs, such as H19, can influence pig muscle development through various mechanisms. Specifically, lncRNA H19 regulates the differentiation of PSCs via distinct pathways (57). H19 exhibits two distinct roles: it serves as a molecular sponge for miR-140-5p, inhibiting PSCs differentiation, and directly binds to the DBN1 protein to regulate their differentiation. Additionally, lncRNA H19 interacts directly with the DNA/RNA-binding protein TDP43 to promote PSCs differentiation (58).

#### 4.1.2 The roles of lncRNAs in myogenesis of cattle

The current NONCODE database^2^ shows that the number of known lncRNAs in cattle is second only to that in pigs, with a total of 23,515. Recent research has extensively identified lncRNAs in bovine muscle tissue. Huang et al. created the first extensive genome-wide catalog of bovine intergenic lncRNAs, identifying a total of 449 lncRNAs situated in intergenic regions (59). Since then, a series of studies have characterized lncRNAs in bovine muscle tissue and explored their functions.

Research has suggested that lncRNAs influence beef quality. Billerey et al. assessed lncRNA expression in *LDM*s and discovered that numerous lncRNAs are positioned within QTLs linked to meat quality (60). Since then, several studies have found that lncRNAs are located in bovine QTL chromosomal regions associated with muscle development (61, 62), and these are mainly linked to those of intramuscular fat (IMF), lean meat, and longissimus muscle regions and are associated with shear forces. One study found that the DELs in hard and tender beef tissues appeared to play important roles in physiological processes associated with meat quality (63). In addition, one study that analyzed bovine and buffalo meat, which had significant differences in shear forces and muscle fiber content, detected 2,161 DELs, which allowed the construction of co-expression and ceRNA networks (64).

Promoting cell proliferation and differentiation can increase meat production. Numerous studies have highlighted the critical role of lncRNAs in regulating the proliferation and differentiation of bovine skeletal muscle. For example, lncRNA H19 facilitates the differentiation of bovine skeletal muscle satellite cells (BSCs) by downregulating myogenic suppressor genes such as *SIRT1* and *FoxO1* (65). LncRNAs have been identified to regulate muscle production in cattle through a variety of pathways. LncRNAs can modulate muscle development in cattle through the adsorption of miRNAs. For instance, Liu et al. discovered and analyzed the lncRNAs in the *LDM* of Shandong Black and Luxi cattle breeds and found that LOC104975788 could compete with miR-133a for binding to *Pax7*. This allowed *Pax7* expression to regulate skeletal muscle development (66). An additional instance is the binding of lncRNA MDNCR (67) to miR-133a, which stimulates *GosB* expression, leading to the differentiation of bovine myoblasts and reducing cell growth. LncRNA-MEG3 interacts with miRNA-135 and *MEF2C* to promote differentiation in bovine skeletal muscle (68). LncRNAs also influence bovine muscle development by modulating the expression of nearby genes, such as lnc403. Lnc403 is specifically expressed in bovine myoblasts and myotubes, where it suppresses the differentiation of BSCs by disrupting the expression of the neighboring gene, *Myf6* (69). In addition, lncRNAs influence muscle development through interactions with proteins. Lnc23 promotes myogenesis in BSCs by binding to PFN1 protein and reducing its inhibitory effect on RhoA and Rac1 (70). Importantly, some lncRNAs such as IGF2 AS can regulate bovine myogenesis through more than one pathway. IGF2 AS acts as a complement to the *IGF2* gene’s intronic area, subsequently influencing the stability and expression levels of *IGF2* mRNA. Additionally, it interacts with the interleukin enhancer binding factor 3 protein to facilitate the proliferation and differentiation of bovine myoblasts.

In addition, some studies have explored lncRNAs in yak muscle. These studies revealed that some lncRNAs had varying expression levels between yaks and bovine-yak hybrids. These lncRNAs could regulate muscle growth and development in bovines through multiple signaling pathways (71). In yak embryos, Ma et al. discovered many DELs in muscle tissues at various developmental stages. Some regulatory elements, like *IGF2* and *Pax7*, were incorporated into the co-expression networks of these lncRNAs along with their corresponding target genes (72). Furthermore, Huang et al. sequenced the transcriptomes of the *LDMs* from hybrids of cattle and yaks at different ages and identified 857 differentially expressed lncRNAs (73). In addition, this group also identified 791 DELs in cattle-yak and yak *LDM* samples and constructed six differentially expressed lncRNA-dominated ceRNA networks (71).

#### 4.1.3 The roles of lncRNAs in myogenesis of sheep

Compared to pigs and cattle, research on lncRNAs in sheep skeletal muscle is less extensive, with only a limited number of lncRNA transcripts identified. However, by analyzing the lncRNAs in the skeletal muscle of sheep at different developmental stages, a total of 4,738 lncRNAs were identified, including 997 that were DELs. Among these, lncGTL2 was highly expressed during the differentiation of skeletal muscle satellite cells (SCs) and was shown to promote myogenic differentiation in sheep by affecting the phosphorylation levels of *PKA* and *CREB* (74). Multiple studies have analyzed lncRNAs in the skeletal muscle of embryos at both gestational and postnatal stages. Li et al. identified 404 lncRNAs with differential expression between prenatal and postnatal stages of sheep skeletal muscle development, providing a detailed expression profile of these lncRNAs in the context of sheep skeletal muscle (75). Yuan et al. also identified several lncRNAs that regulate myogenic differentiation through interactions with miRNAs (76).

At present, research into the roles and mechanisms of lncRNAs in sheep muscle formation is still scarce. However, evidence indicates that lncRNAs could function as miRNA sponges, potentially playing a pivotal role in skeletal muscle development. The lncRNA CTTN-IT1 restores *YAP1* expression by absorbing miR-29a, thereby promoting the proliferation and differentiation of satellite cells in sheep skeletal muscle (77).

#### 4.1.4 The roles of lncRNAs in myogenesis of poultry

Numerous studies, including those by Li et al., have highlighted the critical role of lncRNAs in muscle development in poultry, with Li et al. being the first to identify 281 novel lincRNAs in chicken skeletal muscle at different embryonic stages using RNA-seq technology. Notably, the lncRNA gga-lnc-0181 exhibits high expression levels in skeletal muscle, indicating its potential importance in muscle development (78). Since then, numerous studies have identified lncRNAs in chicken skeletal muscle at different embryonic stages (79–81). Comparing lncRNA expression in skeletal muscle tissues of border chickens with different muscle growth rates at various embryonic stages revealed that specific DELs may be essential in explaining these differences in growth rates (82). Recently, several DELs have been identified at various stages of chicken muscle development. For example, Li et al. investigated lncRNA expression in the *breast* muscles of both juvenile and laying chickens, indicating that these lncRNAs could be involved in breast muscle development via the MAPK signaling pathways (83). Furthermore, Ju et al. also found that lncRNAs play potential regulatory roles in oxidative and glycolytic muscle fibers in chickens (84).

Research has not only identified numerous lncRNAs in chicken muscle through sequencing but has also highlighted their crucial roles in regulating muscle development in poultry. These lncRNAs play a role in multiple processes, including the proliferation and differentiation of myoblasts, muscle fiber differentiation and transformation, and muscle atrophy. Similar to other species, some lncRNAs regulate skeletal muscle development by acting as miRNA sponges. For example, lncRNA-Six1 functions as a ceRNA by binding to miR-1611, thereby modulating the expression of the Six1 protein. This interaction influences both the proliferation and differentiation of chicken myoblasts and the transition between different muscle fiber types (4). LncIRS1 has been demonstrated to regulate muscle mass and fiber composition in living organisms. This lncRNA promotes the proliferation and differentiation of chicken myoblasts *in vitro* by activating the IGF1-PI3K/AKT signaling pathways through adsorption of miR-15 (85). Besides ceRNA mechanisms, some lncRNAs regulate gene expression through both cis and trans interactions. For example, the lncRNA Six1, which is highly expressed in chicken breast tissue, regulates the expression of the *Six1* gene in cis. It encodes a micro-peptide that activates the *Six1* gene, thereby enhancing skeletal muscle cell proliferation and promoting muscle growth in chickens (86). In addition, lncRNAs can interact with proteins to regulate muscle development in chickens. For example, epidermal differentiation protein containing cysteine histidine motifs 1(lncEDCH1) is a lncRNA that shows varying levels of expression between fat broilers and lean Chinese native breeds. The lncEDCH1 acts as a decoy to bind SERCA2 protein in order to increase its stability and its activity. This modulates Ca2+ homeostasis, promotes the slow-twitch phenotype, and helps reduce muscle atrophy (87). Additionally, lncRNA-FKBP1C, found to be differentially expressed between Bai Yinyan (WRR) and Xinghua (XH) chickens, binds to *MYH1B* to stabilize its protein, thereby influencing myoblast proliferation, differentiation, and the transformation of skeletal muscle fiber types (88).

### 4.2 The roles of lncRNAs in lipogenesis of farm animals

Mammalian skeletal muscle is an important aspect of meat quality research, and there is a significant correlation between animal fat deposition and meat production traits. The directional deposition of fat determines the efficiency of feed utilization by farm animals. High subcutaneous fat content leads to poor meat quality, but IMF is crucial for regulating the tenderness, water retention, and flavor of the meat. In recent decades, a growing number of lncRNAs have been recognized for their significant roles in adipose tissue (Table 2). Research indicates that lncRNAs can regulate gene expression and signaling pathways associated with adipogenesis through various mechanisms.

**Table 2:** LncRNA-mediated regulation of adipose tissues.

LncRNAs	Role in adipogenesis	Partner	Species	Reference
ADNCR	As a competitive the endogenous RNA absorbs miR-204, it prevents miR-204 from inhibiting its target gene-histone deacetylase 1 (sirtuin 1, SIRT1), thereby inhibiting adipogenesis.	MiR-204	*Bos taurus*	(110)
MIR221HG	Inhibits the differentiation of bovine adipocytes.	YY1	*Bos taurus*	(107)
BADLNCR1	By negatively regulating the expression of GLRX5 gene, it can inhibit the differentiation of bovine adipocytes.	GLRX5	*Bos taurus*	(7)
CCPG1	As the sponge of miR-93, the inhibitory effect of miR-93 on the expression of lncSLC30A9 is relieved, and lncSLC30A9 promotes adipocyte differentiation by recruiting FOS protein to the promoter of PPARγ.	MiR-93	*Bos taurus*	(8)
ADINR	Activation of C/EBPa transcription and regulation of adipogenesis through histone modification.	C/EBPα	*Homo sapiens*	(141)
Plnc1	Promotes adipocyte differentiation by reducing the methylation level of the CpG region of the PPAR-γ2 promoter and increasing the activity of the PPAR-γ2 promoter.	PPAR-γ2	*Mus musculus*	(142)
NEAT1	The mature miR-140 binds to NEAT1 to increase NEAT1 expression.	MiR-140	*Mus musculus*	(143)
HOXA11-AS1	Regulates adipogenesis by promoting the transcription of adipogenesis-related genes (CEBP-α, DGAT2).	CEBP-α/DGAT2	*Homo sapiens*	(144)
ADAL	Interacts with hnRNPU and IGF2BP2 to regulate adipocyte differentiation and adipogenesis.	HnRNPU/IGF2BP2	*Homo sapiens*	(145)
H19	Inhibits adipogenesis by inhibiting the expression of HDAC in the process of adipogenesis and negatively regulate the expression of Lcor adipogenesis by adsorption of miR-188.	MiR-675/miR-188	*Homo sapiens*	(146)
Uc.417	Inhibits the phosphorylation of p38-MAPK, a key regulator of brown fat activation, to inhibit adipogenesis.	p38-MAPK	*Mus musculus*	(147)
LncBATE1	Combines with hnRNP U to form a functional ribonucleoprotein complex to promote brown fat production.	HnRNP U	*Mus musculus*	(148)
LncBATE10	Increase the expression of PGC1α by competitively binding Celf1 to regulate brown adipogenesis.	PGC1α	*Mus musculus*	(149)
BLNC1	Regulates adipogenesis by forming a ribonucleoprotein complex with EBF2.	EBF2	*Mus musculus*	(150)
SRA	Promote fat differentiation by activating IGF-1 related signaling pathways.	IGF1	*Mus musculus*	(151)
PU.1 AS	Prevent PU.1 translation and promote adipogenesis by forming mRNA/AS lncRNAs duplex with PU.1 mRNA.	PU.1	*Sus scrofa*	(38)
MIR31HG	Promotes adipogenesis by regulating histone modifications at the Fabp4 promoter.	Fabp4	*Homo sapiens*	(152)
Paral1	Regulates adipogenesis by binding to RBM14 to enhance PPARγ transcriptional activity.	PPARγ	*Mus musculus*	(153)
TCONS_00041960	Causes inhibition of adipogenic differentiation by regulating the expression of Gilz as a competitive endogenous RNA of miR-125a-3p.	MiR-204/miR-125a	*Rattus norvegicus*	(154)
LncRNA-Adi	Interacts with microRNA (miR)-449a to enhance cyclin-dependent kinase (CDK)6 expression during adipogenesis.	MiR-449a	*Rattus norvegicus*	(155)
LncRNA-NEF	Inhibition of adipogenesis by modulating the miR-155/PTEN axis.	MiR-155/PTEN axis	*Homo sapiens*	(156)
RP11-142A22.4	Regulation of Wnt5β expression by sponge miR-587 promotes adipogenesis.	MiR-587	*Homo sapiens*	(157)

#### 4.2.1 The roles of lncRNAs in lipogenesis of pig

For the past few years, reports have emerged about the discovery of lncRNAs in porcine intramuscular (PIM) tissue (89–91), dorsal (92, 93), and subcutaneous adipose tissues (94–96). LncRNAs are critical in porcine adipogenesis and can influence processes such as proliferation (97) and differentiation (98) of porcine adipocytes, which in turn affects meat quality. Inadequate IMF and excessive subcutaneous (SC) fat present the primary challenges to pork quality (99).

The content of intramuscular fat was positively associated with the flavor, tenderness, and juiciness of pork and was closely linked to overall pork quality (97, 100). LncRNAs were found to be involved in intramuscular lipogenesis in pigs. Zou et al. conducted transcriptome sequencing of porcine *LDM* at four developmental stages with varying IMF content, and identified 1,032 lincRNAs, among which 6 lincRNAs might be crucial for IMF development (91). Another investigation discovered 6 DELs associated with pathways related to fat deposition and lipid metabolism in the IMF of Jinhua and Landrace pigs (89). Additionally, the differentially expressed lnc_000414 has been shown to inhibit the proliferation of porcine intramuscular adipocytes in fat-type versus lean-type pigs (97).

SC fat accumulation correlates with lean carcass percentage (101); however, excessive deposition of subcutaneous fat can impair growth performance and reduce meat production efficiency (95). LncRNAs can also regulate subcutaneous fat deposition (94–96). For instance, Zhang et al. performed transcriptome sequencing on subcutaneous adipocytes from Jiaxing black pigs and Large White pigs, which exhibit substantial differences in subcutaneous fat deposition. They observed that several DEL target genes were implicated in the PI3K-Akt and MAPK signaling pathways, which are associated with fat formation and lipid metabolism (96).

Adipocyte maturation must go through two important steps: adipocyte proliferation and adipocyte differentiation. Currently, many lncRNAs are considered to be key regulators of porcine adipocyte proliferation and differentiation. For example, lncIMF4 inhibited adipogenesis in PIM pre-adipocytes by promoting lipolysis (102). LncRNAs regulate porcine lipogenesis through various mechanisms, including interactions with other RNAs. For instance, PU.1 AS lncRNAs, transcribed from the porcine *PU.1* gene, can suppress PU.1 protein expression and promote porcine adipogenesis by forming sense-antisense duplexes with *PU.1* mRNA (38). Furthermore, lncRNAs can also interact with proteins to regulate porcine fat development. LncMYOZ2 was shown to interact with adenosylhomocysteinase (AHCY) protein to regulate *MYOZ2* expression and thus promote adipogenesis and differentiation in porcine pre-adipocytes (98). Several lncRNAs are known to regulate adipogenesis by acting as ceRNAs. The lncRNA IMFlnc1 promoted adipogenesis in PIM adipocytes by absorbing miR-199a, thereby upregulating the expression of the *caveolin-1* gene in a similar way to some ceRNAs (37). In addition, lncIMF2 can promote adipogenesis in PIM pre-adipocytes by sponging miR-217 (103).

#### 4.2.2 The roles of lncRNAs in lipogenesis of cattle

Tenderness, flavor, juiciness, and color are crucial parameters for assessing beef quality. The deposition of fat is related to the quality of beef. LncRNAs have been identified as essential regulators of lipogenesis. Recent research has revealed numerous lncRNAs linked to bovine adipose development by analyzing their expression across various developmental stages of adipose tissue (104, 105) and by contrasting them with muscle tissues (64).

The IMF content influences the degree of marbling and is regarded as a key factor impacting the sensory quality of beef (106). However, the function of lncRNAs in intramuscular fat deposition in cattle is not yet fully understood, with only a few studies addressing this aspect. Yang et al. performed comprehensive transcriptional sequencing and analyzed intramuscular preadipocytes at various differentiation stages in Qinchuan cattle, identifying 501 differentially expressed lncRNAs. In addition, they found that the lncRNAs’ target genes are linked to pathways related to lipogenesis and adipocyte differentiation. They proposed that some lncRNAs may absorb miRNAs and regulate lipogenesis (105). Another study identified lncRNAs in the *longest back* muscles of yaks with varying intramuscular fat content and made a similar finding (9). The function of lncRNAs in bovine intramuscular fat still requires further investigation.

Preadipocytes such as fibroblasts gradually develop into adipocytes, and adipocytes continue to accumulate and eventually form adipose tissue within. Adipogenesis occurs through the proliferation and differentiation of adipocytes. LncRNAs play a vital role in regulating the proliferation and differentiation of adipocytes. The novel lncRNA miR-221 host gene (MIR221HG), located in the transcripts of beef cattle, was identified as having differential expression during adipocyte differentiation in beef cattle, and its inhibition significantly increased adipocyte differentiation (107). Furthermore, the expression of lncFAM200B, which had higher levels in fat than in muscles, reduced cyclin D1 expression and notably suppressed the proliferation of bovine pre-adipocytes (108). LncRNAs have been shown to affect both the proliferation and differentiation of bovine adipocytes through multiple pathways. LncRNAs can influence the proliferation and differentiation of bovine adipocytes by cis-regulating gene expression. GLRX5 acts as a stimulator that enhances lipid droplet formation and the expression of adipogenic genes. Conversely, the bovine adipocyte differentiation–related lncRNA 1 (lncRNA BADLNCR1) suppresses bovine lipogenesis by downregulating *GLRX5* expression (7). In addition, lncRNAs can also regulate cattle fat development as ceRNAs. For example, lncPRRX1 functions as a ceRNA to promote bovine myoblast proliferation by releasing CDC42 by competitively binding to miRNA-137 (109). The lncRNA, adipocyte differentiation-associated lncRNA (ADNCR), inhibits adipocyte differentiation by competing with miR-204 for binding, preventing it from inhibiting its target gene, *SIRT1*, which is a histone deacetylase (110). In buffalo adipocytes, lncRNA NDUFC2-AS promotes adipogenic differentiation by increasing the expression of *C/EBP-α* and *THRSP* (111). Sorting and assembly machinery component 50 (LncSAMM50) was also implicated to promote adipogenic differentiation of buffalo adipocytes by upregulating adipogenic markers in a 3 T3-L1 cell line *in vitro* (112). The regulation of lncRNAs in adipose tissue is intricate and encompasses various pathways. Therefore, evaluating their effects on adipocyte proliferation and differentiation requires consideration of their impact on signaling pathways as well.

#### 4.2.3 The roles of lncRNAs in lipogenesis of sheep

At present, research on the regulatory functions of lncRNAs in sheep fat development is limited, with the majority of studies concentrating specifically on tail fat. Some DELs were identified by sequencing adipose tissue from different sheep breeds. Ma et al. performed high-throughput sequencing on tail adipose tissue from sheep breeds with varying levels of tail fat and identified 37 DELs (113). The study revealed that certain lncRNAs are involved in fatty acid metabolism and elongation, as well as in other pathways contributing to fat deposition. Another study identified 7 DELs in fat-tailed versus thin-tailed sheep, with target genes associated with fat development pathways. Notably, three of these lncRNAs were located within the QTLs linked to ‘tail fat deposition,’ indicating their potential role in lipid metabolism (114). Su et al. performed comprehensive transcriptome sequencing on tail tissues from sheep breeds with varying tail types, identifying 728 DELs. Among them, lncRNA-MSTRG.24995 directly affected tail fat deposition through the *FASN* gene, while lncRNA-MSTRG.36913 indirectly affected this process through the *THRSP* gene (115). In addition, Bao et al. investigated a DEL in the *longissimus thoracis* muscle of Tibetan sheep at four different developmental stages. This lncRNA was characterized as a trans-regulator of *FASN* and plays a role in regulating fatty acid metabolism throughout the growth and development of the animals (116). A recent study characterized lncRNAs in sheep intramuscular fat, finding that 61 lncRNAs were differentially expressed during fat deposition in Aohan fine wool sheep. The pathways associated with lipid accumulation were significantly enriched among the target genes of these lncRNAs (117).

#### 4.2.4 The roles of lncRNAs in lipogenesis of poultry

Different from mammalian adipose cells, poultry adipose cells have limited capacity to generate fat. The body fat of broilers is mainly deposited in abdominal fat. Research has demonstrated that lncRNAs can modulate abdominal fat deposition in poultry. Jing et al. performed transcriptome sequencing on the abdominal adipose tissue of both fat and lean broilers, identifying 30 DELs. Among them, 16 lncRNAs were specifically expressed in adipose/lean cells (118).

The first fat to be deposited is intermuscular fat, and the amount of intermuscular fat determines the tenderness of the meat. Research has revealed that lncRNAs can modulate intermuscular fat accumulation in poultry. 7 DELs were identified during the differentiation of intramuscular pre-adipocytes, which may have significant roles in the development of intramuscular pre-adipocytes in chickens (119). LncRNA IMFNCR can also absorb miR-128-3p and miR-27b-3p, which increases the expression of *PPARγ* and promotes the differentiation of chicken intramuscular adipocytes (120).

Many lncRNAs have been discovered to regulate adipogenesis in chickens through various mechanisms. Some lncRNAs can influence gene expression through a *cis*-regulatory mechanism. For instance, adipocyte differentiation-associated lncRNA (lncAD) was identified to promote the expression of *TXNRD1*, thus promoting adipogenic differentiation and inhibiting the proliferation of chicken pre-muscular adipocytes (121). Other studies have also revealed that lncRNAs regulate fat development in chickens at an epigenetic level. For example, Chen et al. sequenced lncRNA in abdominal adipose tissue of broiler strains with different abdominal fat content, and found one DEL, lncPRDM16. The 5′-end functional element of lncPRDM16 is essential for it to inhibit the proliferation of adipocytes and regulate the activity of the *PRDM16* promoter (122). Furthermore, some lncRNAs can also absorb miRNAs and release the expression of their target gene in order to regulate adipose formation in chickens. For example, Tian et al. identified 19,212 potential lncRNAs in the abdominal fat of chickens. MSTRG.25116.1 can absorb miR-1635, leading to increased *FAAH* expression, which is essential for adipogenic differentiation in chicken pre-adipocytes (119). LncRNA FNIP2 has been shown to accelerate chicken lipid synthesis through the release of FNIP2 by adsorption of miR-24-3p (123).

## 5 Discussion and perspectives

In this review, we discuss the latest developments in lncRNA research concerning the biogenesis, myogenesis, and lipogenesis of lncRNAs in farm animals, including pigs, cattle, sheep, and poultry. Our review highlights the growing understanding of lncRNAs and their significant impact on various biological processes affecting farm animal production. Advancements in modern molecular biology and next-generation sequencing technologies have led to the identification of an increasing number of lncRNAs associated with farm animal traits, including muscle and fat development (7–11). However, a comprehensive understanding of lncRNA functions and mechanisms in farm animals remains incomplete, with many lncRNAs and their roles still to be fully elucidated.

A major objective in farm animal production is to enhance meat yield and quality through the regulation of lncRNA expression. This involves developing strategies to harness lncRNAs, which are known to play a vital role in regulating muscle cell proliferation, differentiation, and atrophy. Techniques such as gene editing, RNA interference, and antisense oligonucleotides have been employed to overexpress or knock down specific lncRNAs, thereby influencing muscle hypertrophy and overall meat yield.

However, progress in lncRNA research in farm animals is limited by several factors. Firstly, there is a lack of data regarding the recognition and functional annotation of lncRNAs. Current lncRNA databases primarily cover humans and mice, with only a few providing expression profiles of lncRNAs in farm animals. The lncRNA sequences are generally less conserved among farm animal species, which can lead to recognition impairment due to insertions and deletions within these sequences, which may occur at the same position in the genome. Secondly, the annotation of lncRNAs in farm animals is far less complete than in humans and mice, both regarding the quantity of gene loci and the variety of alternative isomers identified. Therefore, there is a concerted and urgent need to accelerate the annotation of the non-coding regions of the farm animal genome. Thirdly, the focus is primarily on identifying lncRNAs, with a need for further research into their functions and regulatory mechanisms, particularly in cattle and sheep.

Future studies need to explore the functions and mechanisms of action of lncRNAs, as well as clarify their roles in myogenesis, adipogenesis, and other traits in farm animals. In research on the mechanisms of lncRNAs, there are fewer studies on their epigenetic and transcriptional roles, as well as their involvement in pre/during/post transcriptional processes. To explore the mechanisms of lncRNAs more effectively, further development and application of advanced techniques, such as domain-specific chromatin isolation by RNA purification and capture hybridization analysis coupled with RNA target mass spectrometry, are needed. Moreover, existing research on lncRNA functionality is primarily confined to *in vitro* studies involving farm animal cells or cell lines, with a limited number of *in vivo* investigations. Therefore, specific lncRNAs knockout models *in vivo* are required as their effects on the whole organism remain largely unknown. The potential of lncRNA knockout or overexpression for enhancing farm animal breeds still requires further investigation.

In conclusion, lncRNAs represent a promising frontier in farm animal research, with the potential to revolutionize animal breeding and production. The insights gained from studying lncRNAs in myogenesis and lipogenesis offer valuable opportunities for improving farm animal traits. Continued research, supported by technological advancements and interdisciplinary approaches, will be essential for fully realizing the potential benefits of lncRNAs in animal husbandry.

## Footnotes

^1^ http://www.noncode.org/

^2^ http://www.noncode.org/

## References

[1] Zammit PS. Function of the myogenic regulatory factors Myf5, MyoD, Myogenin and MRF4 in skeletal muscle, satellite cells and regenerative myogenesis. Semin Cell Dev Biol. (2017) 72:19–32. doi: 10.1016/j.semcdb.2017.11.011, PMID: 29127046

[2] Dinh TT Blanton JR Jr Riley DG Chase CC Jr Coleman SW Phillips WA. Intramuscular fat and fatty acid composition of longissimus muscle from divergent pure breeds of cattle. J Anim Sci. (2010) 88:756–66. doi: 10.2527/jas.2009-1951, PMID: 19783694

[3] Song C Yang Z Jiang R Cheng J Yue B Wang J. lncRNA IGF2 AS regulates bovine Myogenesis through different pathways. Mol Ther. (2020) 21:874–84. doi: 10.1016/j.omtn.2020.07.002, PMID: 32805490 PMC7452115

[4] Ma M Cai B Jiang L Abdalla BA Li Z Nie Q. lncRNA-Six1 is a target of miR-1611 that functions as a ceRNA to regulate Six1 protein expression and Fiber type switching in chicken Myogenesis. Cells. (2018) 7:243. doi: 10.3390/cells7120243, PMID: 30518151 PMC6315877

[5] Li L Cheng X Chen L Li J Luo W Li C. Long noncoding ribonucleic acid MSTRG.59589 promotes porcine skeletal muscle satellite cells differentiation by enhancing the function of PALLD. Front Genet. (2019) 10:10. doi: 10.3389/fgene.2019.01220, PMID: 31850071 PMC6887656

[6] Cheng X Li L Shi G Chen L Fang C Li M. MEG3 promotes differentiation of porcine satellite cells by sponging miR-423-5p to relieve inhibiting effect on SRF. Cells. (2020) 9:449. doi: 10.3390/cells9020449, PMID: 32075310 PMC7072828

[7] Cai H Li M Jian W Song C Huang Y Lan X. A novel lncRNA BADLNCR1 inhibits bovine adipogenesis by repressing GLRX5 expression. J Cell Mol Med. (2020) 24:7175–86. doi: 10.1111/jcmm.15181, PMID: 32449295 PMC7339203

[8] Kang Z Zhang S Jiang E Wang X Wang Z Chen H. circFLT1 and lncCCPG1 sponges miR-93 to regulate the proliferation and differentiation of adipocytes by promoting lncSLC30A9 expression. Mol Ther. (2020) 22:484–99. doi: 10.1016/j.omtn.2020.09.011, PMID: 33230451 PMC7554329

[9] Wang H Zhong J Zhang C Chai Z Cao H Wang J. The whole-transcriptome landscape of muscle and adipose tissues reveals the ceRNA regulation network related to intramuscular fat deposition in yak. BMC Genomics. (2020) 21:347. doi: 10.1186/s12864-020-6757-z, PMID: 32381004 PMC7203869

[10] Wang Z Yin Z-T Zhang F Li X-Q Chen S-R Yang N. Dynamics of transcriptome changes during subcutaneous preadipocyte differentiation in ducks. BMC Genomics. (2019) 20:6055. doi: 10.1186/s12864-019-6055-9, PMID: 31477016 PMC6720933

[11] Tan Y Gan M Shen L Li L Fan Y Chen Y. Profiling and functional analysis of Long noncoding RNAs and mRNAs during porcine skeletal muscle development. Int J Mol Sci. (2021) 22:503. doi: 10.3390/ijms22020503, PMID: 33419093 PMC7825455

[12] Lv W Jin J Xu Z Luo H Guo Y Wang X. lncMGPF is a novel positive regulator of muscle growth and regeneration. J Cachexia Sarcopenia Muscle. (2020) 11:1723–46. doi: 10.1002/jcsm.12623, PMID: 32954689 PMC7749533

[13] Wei C Wu M Wang C Liu R Zhao H Yang L. Long noncoding RNA Lnc-SEMT modulates IGF2 expression by sponging miR-125b to promote sheep muscle development and growth. Cell Physiol Biochem. (2018) 49:447–62. doi: 10.1159/000492979, PMID: 30153668

[14] Cai B Ma M Yuan R Zhou Z Zhang J Kong S. MYH1G-AS is a chromatin-associated lncRNA that regulates skeletal muscle development in chicken. Cell Mol Biol Lett. (2024) 29:9. doi: 10.1186/s11658-023-00525-x, PMID: 38177995 PMC10765903

[15] Ponting CP Oliver PL Reik W. Evolution and functions of Long noncoding RNAs. Cell. (2009) 136:629–41. doi: 10.1016/j.cell.2009.02.006, PMID: 19239885

[16] Statello L Guo C-J Chen L-L Huarte M. Gene regulation by long non-coding RNAs and its biological functions. Nat Rev Mol Cell Biol. (2020) 22:96–118. doi: 10.1038/s41580-020-00315-9, PMID: 33353982 PMC7754182

[17] Preker P Almvig K Christensen MS Valen E Mapendano CK Sandelin A. PROMoter uPstream transcripts share characteristics with mRNAs and are produced upstream of all three major types of mammalian promoters. Nucleic Acids Res. (2011) 39:7179–93. doi: 10.1093/nar/gkr370, PMID: 21596787 PMC3167610

[18] Wu H Yang L Chen L-L. The diversity of Long noncoding RNAs and their generation. Trends Genet. (2017) 33:540–52. doi: 10.1016/j.tig.2017.05.004, PMID: 28629949

[19] Chen X He L Zhao Y Li Y Zhang S Sun K. Malat1 regulates myogenic differentiation and muscle regeneration through modulating MyoD transcriptional activity. Cell. Discovery. (2017) 3:2017. doi: 10.1038/celldisc.2017.2, PMID: 28326190 PMC5348715

[20] Mattioli K Volders P-J Gerhardinger C Lee JC Maass PG Melé M. High-throughput functional analysis of lncRNA core promoters elucidates rules governing tissue specificity. Genome Res. (2019) 29:344–55. doi: 10.1101/gr.242222.118, PMID: 30683753 PMC6396428

[21] Tsai M-C Manor O Wan Y Mosammaparast N Wang JK Lan F. Long noncoding RNA as modular scaffold of histone modification complexes. Science. (2010) 329:689–93. doi: 10.1126/science.1192002, PMID: 20616235 PMC2967777

[22] Lambrou GI Hatziagapiou K Zaravinos A. The non-coding RNA GAS5 and its role in tumor therapy-induced resistance. Int J Mol Sci. (2020) 21:633. doi: 10.3390/ijms21207633, PMID: 33076450 PMC7588928

[23] Ransohoff JD Wei Y Khavari PA. The functions and unique features of long intergenic non-coding RNA. Nat Rev Mol Cell Biol. (2017) 19:143–57. doi: 10.1038/nrm.2017.104, PMID: 29138516 PMC5889127

[24] Rinn JL Kertesz M Wang JK Squazzo SL Xu X Brugmann SA. Functional demarcation of active and silent chromatin domains in human HOX loci by noncoding RNAs. Cell. (2007) 129:1311–23. doi: 10.1016/j.cell.2007.05.022, PMID: 17604720 PMC2084369

[25] Kelley RL Kuroda MI. Noncoding RNA genes in dosage compensation and imprinting. Cell. (2000) 103:9–12. doi: 10.1016/S0092-8674(00)00099-4, PMID: 11051542

[26] Chen CK Blanco M Jackson C Aznauryan E Ollikainen N Surka C. Xist recruits the X chromosome to the nuclear lamina to enable chromosome-wide silencing. Science. (2016) 354:468–72. doi: 10.1126/science.aae0047, PMID: 27492478

[27] Wang KC Yang YW Liu B Sanyal A Corces-Zimmerman R Chen Y. A long noncoding RNA maintains active chromatin to coordinate homeotic gene expression. Nature. (2011) 472:120–4. doi: 10.1038/nature09819, PMID: 21423168 PMC3670758

[28] Xiang J-F Yin Q-F Chen T Zhang Y Zhang X-O Wu Z. Human colorectal cancer-specific CCAT1-L lncRNA regulates long-range chromatin interactions at the MYC locus. Cell Res. (2014) 24:513–31. doi: 10.1038/cr.2014.35, PMID: 24662484 PMC4011346

[29] Yang Z Xu F Teschendorff AE Zhao Y Yao L Li J. Insights into the role of long non-coding RNAs in DNA methylation mediated transcriptional regulation. Front Mol Biosci. (2022) 9:9. doi: 10.3389/fmolb.2022.1067406, PMID: 36533073 PMC9755597

[30] Martens JA Laprade L Winston F. Intergenic transcription is required to repress the *Saccharomyces cerevisiae* SER3 gene. Nature. (2004) 429:571–4. doi: 10.1038/nature02538, PMID: 15175754

[31] Feng J Bi C Clark BS Mady R Shah P Kohtz JD. The Evf-2 noncoding RNA is transcribed from the Dlx-5/6 ultraconserved region and functions as a Dlx-2 transcriptional coactivator. Genes Dev. (2006) 20:1470–84. doi: 10.1101/gad.1416106, PMID: 16705037 PMC1475760

[32] Postepska-Igielska A Giwojna A Gasri-Plotnitsky L Schmitt N Dold A Ginsberg D. LncRNA Khps1 regulates expression of the proto-oncogene SPHK1 via triplex-mediated changes in chromatin structure. Mol Cell. (2015) 60:626–36. doi: 10.1016/j.molcel.2015.10.001, PMID: 26590717

[33] Engreitz JM Haines JE Perez EM Munson G Chen J Kane M. Local regulation of gene expression by lncRNA promoters, transcription and splicing. Nature. (2016) 539:452–5. doi: 10.1038/nature20149, PMID: 27783602 PMC6853796

[34] Paralkar Vikram R Taborda Cristian C Huang P Yao Y Kossenkov Andrew V Prasad R. Unlinking an lncRNA from its associated cis element. Mol Cell. (2016) 62:104–10. doi: 10.1016/j.molcel.2016.02.029, PMID: 27041223 PMC4877494

[35] Tripathi V Ellis JD Shen Z Song DY Pan Q Watt AT. The nuclear-retained noncoding RNA MALAT1 regulates alternative splicing by modulating SR splicing factor phosphorylation. Mol Cell. (2010) 39:925–38. doi: 10.1016/j.molcel.2010.08.011, PMID: 20797886 PMC4158944

[36] Gong C Li Z Ramanujan K Clay I Zhang Y Lemire-Brachat S. A Long non-coding RNA, LncMyoD, regulates skeletal muscle differentiation by blocking IMP2-mediated mRNA translation. Dev Cell. (2015) 34:181–91. doi: 10.1016/j.devcel.2015.05.009, PMID: 26143994

[37] Wang J Chen M-y Chen J-f Ren Q-l Zhang J-q Cao H. LncRNA IMFlnc1 promotes porcine intramuscular adipocyte adipogenesis by sponging miR-199a-5p to up-regulate CAV-1. BMC Mol Cell Biol. (2020) 21:77. doi: 10.1186/s12860-020-00324-8, PMID: 33148167 PMC7640402

[38] Wei N Wang Y Xu RX Wang GQ Xiong Y Yu TY. PU.1antisense lncRNA against its mRNA translation promotes adipogenesis in porcine preadipocytes. Anim Genet. (2015) 46:133–40. doi: 10.1111/age.12275, PMID: 25691151

[39] Andrews SJ Rothnagel JA. Emerging evidence for functional peptides encoded by short open reading frames. Nat Rev Genet. (2014) 15:193–204. doi: 10.1038/nrg3520, PMID: 24514441

[40] Matsumoto A Pasut A Matsumoto M Yamashita R Fung J Monteleone E. mTORC1 and muscle regeneration are regulated by the LINC00961-encoded SPAR polypeptide. Nature. (2017) 541:228–32. doi: 10.1038/nature21034, PMID: 28024296

[41] Anderson Douglas M Anderson Kelly M Chang C-L Makarewich Catherine A Nelson Benjamin R McAnally John R. A micropeptide encoded by a putative Long noncoding RNA regulates muscle performance. Cell. (2015) 160:595–606. doi: 10.1016/j.cell.2015.01.009, PMID: 25640239 PMC4356254

[42] Nelson BR Makarewich CA Anderson DM Winders BR Troupes CD Wu F. A peptide encoded by a transcript annotated as long noncoding RNA enhances SERCA activity in muscle. Science. (2016) 351:271–5. doi: 10.1126/science.aad4076, PMID: 26816378 PMC4892890

[43] Gao PF Guo XH du M Cao GQ Yang QC Pu ZD. LncRNA profiling of skeletal muscles in large white pigs and Mashen pigs during development1,2. J Anim Sci. (2017) 95:4239–50. doi: 10.2527/jas2016.1297, PMID: 29108073

[44] Hou X Wang L Zhao F Liu X Gao H Shi L. Genome-wide expression profiling of mRNAs, lncRNAs and circRNAs in skeletal muscle of two different pig breeds. Animals. (2021) 11:169. doi: 10.3390/ani11113169, PMID: 34827901 PMC8614396

[45] Huang Z Li Q Li M Li C. Transcriptome analysis reveals the long intergenic noncoding RNAs contributed to skeletal muscle differences between Yorkshire and Tibetan pig. Sci Rep. (2021) 11:126. doi: 10.1038/s41598-021-82126-2, PMID: 33514792 PMC7846844

[46] Sun J Xie M Huang Z Li H Chen T Sun R. Integrated analysis of non-coding RNA and mRNA expression profiles of 2 pig breeds differing in muscle traits. J Anim Sci. (2017) 95:867. doi: 10.2527/jas2016.0867, PMID: 28380516

[47] Zhao WJ Li ZJ Liu Q Xie S Li MX Wang Y. Analysis of long intergenic non-coding RNAs transcriptomic profiling in skeletal muscle growth during porcine embryonic development. Sci Rep. (2021) 11:14. doi: 10.1038/s41598-021-94014-w, PMID: 34315913 PMC8316452

[48] Chen G Cheng X Shi G Zou C Chen L Li J. Transcriptome analysis reveals the effect of Long intergenic noncoding RNAs on pig muscle growth and fat deposition. Biomed Res Int. (2019) 2019:1–15. doi: 10.1155/2019/2951427, PMID: 31341893 PMC6614983

[49] Zou C Li JX Luo WZ Li L Hu A Fu YH. Transcriptome analysis reveals long intergenic non-coding RNAs involved in skeletal muscle growth and development in pig. Sci Rep. (2017) 7:8704. doi: 10.1038/s41598-017-07998-9, PMID: 28821716 PMC5562803

[50] Wang D Pu Y Li Y Pan D Wang S Tian W. Comprehensive analysis of lncRNAs involved in skeletal muscle development in ZBED6-knockout Bama pigs. BMC Genomics. (2021) 22:593. doi: 10.1186/s12864-021-07881-y, PMID: 34348644 PMC8340374

[51] Lee SH Joo ST Ryu YC. Skeletal muscle fiber type and myofibrillar proteins in relation to meat quality. Meat Sci. (2010) 86:166–70. doi: 10.1016/j.meatsci.2010.04.040, PMID: 20605337

[52] Li R Li B Jiang A Cao Y Hou L Zhang Z. Exploring the lncRNAs related to skeletal muscle Fiber types and meat quality traits in pigs. Genes. (2020) 11:883. doi: 10.3390/genes11080883, PMID: 32759632 PMC7465969

[53] Bernat JL Ochoa JL. Muscle hypertrophy after partial denervation: a human case. J Neurol Neurosurg Psychiatry. (1978) 41:719–25. doi: 10.1136/jnnp.41.8.719, PMID: 681959 PMC1083388

[54] Walters J. Muscle hypertrophy and pseudohypertrophy. Pract Neurol. (2017) 17:369–79. doi: 10.1136/practneurol-2017-001695, PMID: 28778933

[55] Lv W Jiang W Luo H Tong Q Niu X Liu X. Long noncoding RNAlncMREFpromotes myogenic differentiation and muscle regeneration by interacting with the Smarca5/p300 complex. Nucleic Acids Res. (2022) 50:10733–55. doi: 10.1093/nar/gkac854 36200826 PMC9561262

[56] Jin J du M Wang J Guo Y Zhang J Zuo H. Conservative analysis of Synaptopodin-2 intron sense-overlapping lncRNA reveals its novel function in promoting muscle atrophy. J Cachexia Sarcopenia Muscle. (2022) 13:2017–30. doi: 10.1002/jcsm.13012, PMID: 35592920 PMC9397560

[57] Li J Su T Zou C Luo W Shi G Chen L. Long non-coding RNA H19 regulates porcine satellite cell differentiation through miR-140-5p/SOX4 and DBN1. Front Cell Dev Biol. (2020) 8:8. doi: 10.3389/fcell.2020.518724, PMID: 33324629 PMC7723966

[58] Li J Zhao W Li Q Huang Z Shi G Li C. Long non-coding RNA H19 promotes porcine satellite cell differentiation by interacting with TDP43. Genes. (2020) 11:259. doi: 10.3390/genes11030259, PMID: 32121115 PMC7140797

[59] Huang W Long N Khatib H. Genome-wide identification and initial characterization of bovine long non-coding RNAs from EST data. Anim Genet. (2012) 43:674–82. doi: 10.1111/j.1365-2052.2012.02325.x, PMID: 22497321

[60] Billerey C Boussaha M Esquerré D Rebours E Djari A Meersseman C. Identification of large intergenic non-coding RNAs in bovine muscle using next-generation transcriptomic sequencing. BMC Genomics. (2014) 15:499. doi: 10.1186/1471-2164-15-499, PMID: 24948191 PMC4073507

[61] Choi J-Y Shin D Lee H-J Oh J-D. Comparison of long noncoding RNA between muscles and adipose tissues in Hanwoo beef cattle. Anim Cells Syst. (2018) 23:50–8. doi: 10.1080/19768354.2018.1512522, PMID: 30834159 PMC6394308

[62] Liu XF Ding XB Li X Jin CF Yue YW Li GP. An atlas and analysis of bovine skeletal muscle long noncoding RNAs. Anim Genet. (2017) 48:278–86. doi: 10.1111/age.12539, PMID: 28262958

[63] Muniz MMM Simielli Fonseca LF Scalez DCB Vega AS Silva DBS Ferro JA. Characterization of novel lncRNA muscle expression profiles associated with meat quality in beef cattle. Evol Appl. (2022) 15:706–18. doi: 10.1111/eva.13365, PMID: 35505883 PMC9046762

[64] Li H Huang K Wang P Feng T Shi D Cui K. Comparison of Long non-coding RNA expression profiles of cattle and Buffalo differing in muscle characteristics. Front Genet. (2020) 11:98. doi: 10.3389/fgene.2020.00098, PMID: 32174968 PMC7054449

[65] Xu X Ji S Li W Yi B Li H Zhang H. LncRNA H19 promotes the differentiation of bovine skeletal muscle satellite cells by suppressing Sirt1/FoxO1. Cell Mol Biol Lett. (2017) 22:10. doi: 10.1186/s11658-017-0040-6, PMID: 28652859 PMC5481879

[66] Liu R Han M Liu X Yu K Bai X Dong Y. Genome-wide identification and characterization of Long non-coding RNAs in longissimus dorsi skeletal muscle of Shandong black cattle and Luxi cattle. Front Genet. (2022) 13:399. doi: 10.3389/fgene.2022.849399, PMID: 35651943 PMC9149217

[67] Li H Yang J Jiang R Wei X Song C Huang Y. Long non-coding RNA profiling reveals an abundant MDNCR that promotes differentiation of myoblasts by sponging miR-133a. Mol Ther. (2018) 12:610–25. doi: 10.1016/j.omtn.2018.07.003, PMID: 30195797 PMC6078111

[68] Liu M Li B Peng W Ma Y Huang Y Lan X. LncRNA-MEG3 promotes bovine myoblast differentiation by sponging miR-135. J Cell Physiol. (2019) 234:18361–70. doi: 10.1002/jcp.28469, PMID: 30887511

[69] Zhang XJ Chen MM Liu XF Zhang LL Ding XB Guo YW. A novel lncRNA, lnc403, involved in bovine skeletal muscle myogenesis by mediating KRAS/Myf6. Gene. (2020) 751:144706. doi: 10.1016/j.gene.2020.144706, PMID: 32387386

[70] Chen M Zhang L Guo Y Liu X Song Y Li X. A novel lncRNA promotes myogenesis of bovine skeletal muscle satellite cells via PFN1-RhoA/Rac1. J Cell Mol Med. (2021) 25:5988–6005. doi: 10.1111/jcmm.16427, PMID: 33942976 PMC8256363

[71] Huang C Ge F Ma X Dai R Dingkao R Zhaxi Z. Comprehensive analysis of mRNA, lncRNA, circRNA, and miRNA expression profiles and their ceRNA networks in the longissimus Dorsi muscle of cattle-yak and yak. Front Genet. (2021) 12:557. doi: 10.3389/fgene.2021.772557, PMID: 34966412 PMC8710697

[72] Ma X Fu D Chu M Ding X Wu X Guo X. Genome-wide analysis reveals changes in polled yak Long non-coding RNAs in skeletal muscle development. Front Genet. (2020) 11:365. doi: 10.3389/fgene.2020.00365, PMID: 32351548 PMC7176074

[73] Huang C Dai R Meng G Dingkao R Wang X Ren W. Transcriptome-wide study of mRNAs and lncRNAs modified by m6A RNA methylation in the longissimus Dorsi muscle development of cattle-yak. Cells. (2022) 11:654. doi: 10.3390/cells11223654, PMID: 36429081 PMC9688506

[74] Chen Q Bao J-J Zhang H-C Huang C Zhao Q. LncRNA GTL2 regulates myoblast proliferation and differentiation via the PKA-CREB pathway in Duolang sheep. Zool Res. (2024) 45:1261–75. doi: 10.24272/j.issn.2095-8137.2024.125, PMID: 39397245 PMC11668951

[75] Li C-Y Li X Liu Z Ni W Zhang X Hazi W. Identification and characterization of long non-coding RNA in prenatal and postnatal skeletal muscle of sheep. Genomics. (2019) 111:133–41. doi: 10.1016/j.ygeno.2018.01.009, PMID: 29366530

[76] Yuan C Zhang K Yue Y Guo T Liu J Niu C. Analysis of dynamic and widespread lncRNA and miRNA expression in fetal sheep skeletal muscle. PeerJ. (2020) 8:e9957. doi: 10.7717/peerj.9957, PMID: 33024632 PMC7518186

[77] Wu T Wang S Wang L Zhang W Chen W Lv X. Long noncoding RNA (lncRNA) CTTN-IT1 elevates skeletal muscle satellite cell proliferation and differentiation by acting as ceRNA for YAP1 through absorbing miR-29a in Hu sheep. Front Genet. (2020) 11:843. doi: 10.3389/fgene.2020.00843, PMID: 32849826 PMC7427492

[78] Li T Wang S Wu R Zhou X Zhu D Zhang Y. Identification of long non-protein coding RNAs in chicken skeletal muscle using next generation sequencing. Genomics. (2012) 99:292–8. doi: 10.1016/j.ygeno.2012.02.003, PMID: 22374175

[79] Li Y Jin W Zhai B Chen Y Li G Zhang Y. LncRNAs and their regulatory networks in breast muscle tissue of Chinese Gushi chickens during late postnatal development. BMC Genomics. (2021) 22:356. doi: 10.1186/s12864-020-07356-6, PMID: 33422015 PMC7797159

[80] Li Z Ouyang H Zheng M Cai B Han P Abdalla BA. Integrated analysis of Long non-coding RNAs (LncRNAs) and mRNA expression profiles reveals the potential role of LncRNAs in skeletal muscle development of the chicken. Front Physiol. (2017) 7:687. doi: 10.3389/fphys.2016.00687, PMID: 28119630 PMC5220077

[81] Pan Z Yang C Zhao R Jiang X Yu C Li Z. Characterization of lncRNA/circRNA-miRNA-mRNA network to reveal potential functional ceRNAs in the skeletal muscle of chicken. Front Physiol. (2022) 13:854. doi: 10.3389/fphys.2022.969854, PMID: 36246144 PMC9558166

[82] Wu P Zhou K Zhang J Ling X Zhang X Li P. Transcriptome integration analysis at different embryonic ages reveals key lncRNAs and mRNAs for chicken skeletal muscle. Frontiers in veterinary. Science. (2022) 9:9. doi: 10.3389/fvets.2022.908255, PMID: 35782545 PMC9244430

[83] Loor JJ Li D Li F Jiang K Zhang M Han R. Integrative analysis of long noncoding RNA and mRNA reveals candidate lncRNAs responsible for meat quality at different physiological stages in Gushi chicken. PLoS One. (2019) 14:5006. doi: 10.1371/journal.pone.0215006, PMID: 30964907 PMC6456248

[84] Ju X Liu Y Shan Y Ji G Zhang M Tu Y. Analysis of potential regulatory LncRNAs and CircRNAs in the oxidative myofiber and glycolytic myofiber of chickens. Sci Rep. (2021) 11:20861. doi: 10.1038/s41598-021-00176-y, PMID: 34675224 PMC8531282

[85] Li Z Cai B Abdalla BA Zhu X Zheng M Han P. LncIRS1 controls muscle atrophy via sponging miR-15 family to activate IGF1-PI3K/AKT pathway. J Cachexia Sarcopenia Muscle. (2019) 10:391–410. doi: 10.1002/jcsm.12374, PMID: 30701698 PMC6463472

[86] Cai B Li Z Ma M Wang Z Han P Abdalla BA. LncRNA-Six1 encodes a micropeptide to activate Six1 in Cis and is involved in cell proliferation and muscle growth. Front Physiol. (2017) 8:230. doi: 10.3389/fphys.2017.00230, PMID: 28473774 PMC5397475

[87] Cai B Ma M Zhang J Wang Z Kong S Zhou Z. LncEDCH1 improves mitochondrial function to reduce muscle atrophy by interacting with SERCA2. Mol Ther. (2022) 27:319–34. doi: 10.1016/j.omtn.2021.12.004, PMID: 35024244 PMC8717430

[88] Yu J-a Wang Z Yang X Ma M Li Z Nie Q. LncRNA-FKBP1C regulates muscle fiber type switching by affecting the stability of MYH1B. Cell death. Discovery. (2021) 7:463. doi: 10.1038/s41420-021-00463-7, PMID: 33837177 PMC8035166

[89] Miao Z Wang S Zhang J Wei P Guo L Liu D. Identification and comparison of long non-conding RNA in Jinhua and landrace pigs. Biochem Biophys Res Commun. (2018) 506:765–71. doi: 10.1016/j.bbrc.2018.06.028, PMID: 29890140

[90] Zappaterra M Tan L Chen Z Teng M Chen B Xu H. Genome-wide analysis of mRNAs, lncRNAs, and circRNAs during intramuscular adipogenesis in Chinese Guizhou Congjiang pigs. PLoS One. (2022) 17:1293. doi: 10.1371/journal.pone.0261293, PMID: 35077458 PMC8789167

[91] Zou C Li L Cheng X Li C Fu Y Fang C. Identification and functional analysis of Long intergenic non-coding RNAs underlying intramuscular fat content in pigs. Front Genet. (2018) 9:102. doi: 10.3389/fgene.2018.00102, PMID: 29662503 PMC5890112

[92] Kumar H Srikanth K Park W Lee S-H Choi B-H Kim H. Transcriptome analysis to identify long non coding RNA (lncRNA) and characterize their functional role in back fat tissue of pig. Gene. (2019) 703:71–82. doi: 10.1016/j.gene.2019.04.014, PMID: 30954676

[93] Xing K Wang K Ao H Chen S Tan Z Wang Y. Comparative adipose transcriptome analysis digs out genes related to fat deposition in two pig breeds. Sci Rep. (2019) 9:12925. doi: 10.1038/s41598-019-49548-5, PMID: 31501489 PMC6733950

[94] Feng H Liu T Yousuf S Zhang X Huang W Li A. Identification and analysis of lncRNA, miRNA and mRNA related to subcutaneous and intramuscular fat in Laiwu pigs. Front Endocrinol. (2023) 13:1460. doi: 10.3389/fendo.2022.1081460, PMID: 36714570 PMC9880541

[95] Liu X Liu K Shan B Wei S Li D Han H. A genome-wide landscape of mRNAs, lncRNAs, and circRNAs during subcutaneous adipogenesis in pigs. J Anim Sci Biotechnol. (2018) 9:76. doi: 10.1186/s40104-018-0292-7, PMID: 30410752 PMC6211446

[96] Zhang D Wu W Huang X Xu K Zheng C Zhang J. Comparative analysis of gene expression profiles in differentiated subcutaneous adipocytes between Jiaxing black and large white pigs. BMC Genomics. (2021) 22:361. doi: 10.1186/s12864-020-07361-9, PMID: 33468065 PMC7814706

[97] Sun Y Chen X Qin J Liu S Zhao R Yu T. Comparative analysis of Long noncoding RNAs expressed during intramuscular adipocytes Adipogenesis in fat-type and lean-type pigs. J Agric Food Chem. (2018) 66:12122–30. doi: 10.1021/acs.jafc.8b04243, PMID: 30339027

[98] Yang Y Wu Y Ji M Rong X Zhang Y Yang S. The long non-coding RNA lncMYOZ2 mediates an AHCY/MYOZ2 axis to promote adipogenic differentiation in porcine preadipocytes. BMC Genomics. (2022) 23:700. doi: 10.1186/s12864-022-08923-9, PMID: 36221052 PMC9552422

[99] Wu W Zhang D Yin Y Ji M Xu K Huang X. Comprehensive transcriptomic view of the role of the LGALS12 gene in porcine subcutaneous and intramuscular adipocytes. BMC Genomics. (2019) 20:509. doi: 10.1186/s12864-019-5891-y, PMID: 31215398 PMC6582507

[100] Huang W Zhang X Li A Xie L Miao X. Genome-wide analysis of mRNAs and lncRNAs of intramuscular fat related to lipid metabolism in two pig breeds. Cell Physiol Biochem. (2018) 50:2406–22. doi: 10.1159/000495101, PMID: 30423578

[101] Grunert KG Bredahl L Brunsø K. Consumer perception of meat quality and implications for product development in the meat sector—a review. Meat Sci. (2004) 66:259–72. doi: 10.1016/S0309-1740(03)00130-X, PMID: 22064127

[102] Sun Y Cai R Wang Y Zhao R Qin J Pang W. A newly identified LncRNA LncIMF4 controls Adipogenesis of porcine intramuscular Preadipocyte through attenuating autophagy to inhibit lipolysis. Animals. (2020) 10:926. doi: 10.3390/ani10060926, PMID: 32466602 PMC7341528

[103] Yi X He Z Tian T Kou Z Pang W. LncIMF2 promotes adipogenesis in porcine intramuscular preadipocyte through sponging MiR-217. Anim Biotechnol. (2023) 34:268–79. doi: 10.1080/10495398.2021.1956509, PMID: 34346296

[104] Jiang R Li H Huang Y Lan X Lei C Chen H. Transcriptome profiling of lncRNA related to fat tissues of Qinchuan cattle. Gene. (2020) 742:144587. doi: 10.1016/j.gene.2020.144587, PMID: 32179170

[105] Yang X Ma X Mei C Zan L. A genome-wide landscape of mRNAs, lncRNAs, circRNAs and miRNAs during intramuscular adipogenesis in cattle. BMC Genomics. (2022) 23:691. doi: 10.1186/s12864-022-08911-z, PMID: 36203142 PMC9535873

[106] Stewart SM Gardner GE McGilchrist P Pethick DW Polkinghorne R Thompson JM. Prediction of consumer palatability in beef using visual marbling scores and chemical intramuscular fat percentage. Meat Sci. (2021) 181:108322. doi: 10.1016/j.meatsci.2020.108322, PMID: 33067083

[107] Li M Gao Q Tian Z Lu X Sun Y Chen Z. MIR221HG is a novel Long noncoding RNA that inhibits bovine adipocyte differentiation. Genes. (2019) 11:29. doi: 10.3390/genes11010029, PMID: 31887993 PMC7016960

[108] Zhang S Kang Z Cai H Jiang E Pan C Dang R. Identification of novel alternative splicing of bovine lncRNA lncFAM200B and its effects on preadipocyte proliferation. J Cell Physiol. (2020) 236:601–11. doi: 10.1002/jcp.29887, PMID: 32542663

[109] Zhang W Sun B Zhao Y Raza SHA Li Y Wang J. Proliferation of bovine myoblast by LncPRRX1 via regulation of the miR-137/CDC42 axis. Int J Biol Macromol. (2022) 220:33–42. doi: 10.1016/j.ijbiomac.2022.08.018, PMID: 35944756

[110] Li M Sun X Cai H Sun Y Plath M Li C. Long non-coding RNA ADNCR suppresses adipogenic differentiation by targeting miR-204. Biochim Biophys. (2016) 1859:871–82. doi: 10.1016/j.bbagrm.2016.05.003, PMID: 27156885

[111] Huang J Zheng Q Wang S Wei X Li F Ma Y. High-throughput RNA sequencing reveals NDUFC2-AS lncRNA promotes Adipogenic differentiation in Chinese Buffalo (*Bubalus bubalis* L.). Genes. (2019) 10:689. doi: 10.3390/genes10090689, PMID: 31500202 PMC6770997

[112] Zhu R Feng X Wei Y Guo D Li J Liu Q. lncSAMM50 enhances Adipogenic differentiation of Buffalo adipocytes with no effect on its host gene. Front Genet. (2021) 12:158. doi: 10.3389/fgene.2021.626158, PMID: 33841496 PMC8033173

[113] Ma L Zhang M Jin Y Erdenee S Hu L Chen H. Comparative transcriptome profiling of mRNA and lncRNA related to tail adipose tissues of sheep. Front Genet. (2018) 9:365. doi: 10.3389/fgene.2018.00365, PMID: 30250481 PMC6139350

[114] Bakhtiarizadeh MR Salami SA. Identification and expression analysis of Long noncoding RNAs in fat-tail of sheep breeds. G3. (2019) 9:1263–76. doi: 10.1534/g3.118.201014, PMID: 30787031 PMC6469412

[115] Su XH He HY Fang C Liu LL Liu WJ. Transcriptome profiling of LncRNAs in sheep tail fat deposition. Anim Biotechnol. (2023) 34:900–10. doi: 10.1080/10495398.2021.2002882, PMID: 34865605

[116] Bao G Li S Zhao F Wang J Liu X Hu J. Comprehensive transcriptome analysis reveals the role of lncRNA in fatty acid metabolism in the longissimus Thoracis muscle of Tibetan sheep at different ages. Frontiers. Nutrition. (2022) 9:9. doi: 10.3389/fnut.2022.847077, PMID: 35369085 PMC8964427

[117] Han F Li J Zhao R Liu L Li L Li Q. Identification and co-expression analysis of long noncoding RNAs and mRNAs involved in the deposition of intramuscular fat in Aohan fine-wool sheep. BMC Genomics. (2021) 22:98. doi: 10.1186/s12864-021-07385-9, PMID: 33526009 PMC7852088

[118] Jing Y Cheng B Wang H Bai X Zhang Q Wang N. The landscape of the long non-coding RNAs and circular RNAs of the abdominal fat tissues in the chicken lines divergently selected for fatness. BMC Genomics. (2022) 23:45. doi: 10.1186/s12864-022-09045-y, PMID: 36456907 PMC9714206

[119] Tian W Hao X Nie R Ling Y Zhang B Zhang H. Comparative transcriptome analysis reveals regulatory mechanism of Long non-coding RNAs during abdominal Preadipocyte Adipogenic differentiation in chickens. Animals. (2022) 12:1099. doi: 10.3390/ani12091099, PMID: 35565526 PMC9101879

[120] Zhang M Li F Sun J-w Li D-h Li W-t Jiang R-r. LncRNA IMFNCR promotes intramuscular adipocyte differentiation by sponging miR-128-3p and miR-27b-3p. Front Genet. (2019) 10:10. doi: 10.3389/fgene.2019.00042, PMID: 30804984 PMC6378276

[121] Zhang M Ma X Zhai Y Zhang D Sui L Li W. Comprehensive transcriptome analysis of lncRNAs reveals the role of lncAD in chicken intramuscular and abdominal Adipogenesis. J Agric Food Chem. (2020) 68:3678–88. doi: 10.1021/acs.jafc.9b07405, PMID: 32125837

[122] Chen Y Zhao S Ding R Li H Yang C-X Du Z-Q. Identification of a Long noncoding RNA (lncPRDM16) inhibiting Preadipocyte proliferation in the chicken. J Agric Food Chem. (2022) 70:1335–45. doi: 10.1021/acs.jafc.1c05554, PMID: 35048701

[123] Guo L Chao X Huang W Li Z Luan K Ye M. Whole transcriptome analysis reveals a potential regulatory mechanism of LncRNA-FNIP2/miR-24-3p/FNIP2 Axis in chicken Adipogenesis. Front Cell Dev Biol. (2021) 9:9. doi: 10.3389/fcell.2021.653798, PMID: 34249911 PMC8265275

[124] Wang L Zhao Y Bao X Zhu X Kwok YK Sun K. LncRNA Dum interacts with Dnmts to regulate Dppa2 expression during myogenic differentiation and muscle regeneration. Cell Res. (2015) 25:335–50. doi: 10.1038/cr.2015.21, PMID: 25686699 PMC4349245

[125] Zhou L Sun K Zhao Y Zhang S Wang X Li Y. Linc-YY1 promotes myogenic differentiation and muscle regeneration through an interaction with the transcription factor YY1. Nat Commun. (2015) 6:10026. doi: 10.1038/ncomms10026, PMID: 26658965

[126] Zhou Y Cheunsuchon P Nakayama Y Lawlor MW Zhong Y Rice KA. Activation of paternally expressed genes and perinatal death caused by deletion of theGtl2gene. Development. (2010) 137:2643–52. doi: 10.1242/dev.045724, PMID: 20610486 PMC2910384

[127] Yu X Zhang Y Li T Ma Z Jia H Chen Q. Long non-coding RNA Linc-RAM enhances myogenic differentiation by interacting with MyoD. Nature. Communications. (2017) 8:16. doi: 10.1038/ncomms14016, PMID: 28091529 PMC5241866

[128] Caretti G Schiltz RL Dilworth FJ di M Zhao P Ogryzko V. The RNA helicases p68/p72 and the noncoding RNA SRA are Coregulators of MyoD and skeletal muscle differentiation. Dev Cell. (2006) 11:547–60. doi: 10.1016/j.devcel.2006.08.003, PMID: 17011493

[129] Mueller AC Cichewicz MA Dey BK Layer R Reon BJ Gagan JR. MUNC, a Long noncoding RNA that facilitates the function of MyoD in skeletal Myogenesis. Mol Cell Biol. (2023) 35:498–513. doi: 10.1128/MCB.01079-14, PMID: 25403490 PMC4285423

[130] Mousavi K Zare H Dell’Orso S Grontved L Gutierrez-Cruz G Derfoul A. eRNAs promote transcription by establishing chromatin accessibility at defined genomic loci. Mol Cell. (2013) 51:606–17. doi: 10.1016/j.molcel.2013.07.022, PMID: 23993744 PMC3786356

[131] Cesana M Cacchiarelli D Legnini I Santini T Sthandier O Chinappi M. A Long noncoding RNA controls muscle differentiation by functioning as a competing endogenous RNA. Cell. (2011) 147:358–69. doi: 10.1016/j.cell.2011.09.028, PMID: 22000014 PMC3234495

[132] Kallen Amanda N Zhou X-B Xu J Qiao C Ma J Yan L. The imprinted H19 LncRNA antagonizes Let-7 MicroRNAs. Mol Cell. (2013) 52:101–12. doi: 10.1016/j.molcel.2013.08.027, PMID: 24055342 PMC3843377

[133] Zhu M Liu J Xiao J Yang L Cai M Shen H. Lnc-mg is a long non-coding RNA that promotes myogenesis. Nature. Communications. (2017) 8:718. doi: 10.1038/ncomms14718, PMID: 28281528 PMC5353601

[134] Sun X Li M Sun Y Cai H Lan X Huang Y. The developmental transcriptome sequencing of bovine skeletal muscle reveals a long noncoding RNA, lncMD, promotes muscle differentiation by sponging miR-125b. Biochim Biophys Acta Rev Cancer. (2016) 1863:2835–45. doi: 10.1016/j.bbamcr.2016.08.014, PMID: 27589905

[135] Lu L Sun K Chen X Zhao Y Wang L Zhou L. Genome-wide survey by ChIP-seq reveals YY1 regulation of lincRNAs in skeletal myogenesis. EMBO J. (2013) 32:2575–88. doi: 10.1038/emboj.2013.182, PMID: 23942234 PMC3791367

[136] Wang J Gong C Maquat LE. Control of myogenesis by rodent SINE-containing lncRNAs. Genes Dev. (2013) 27:793–804. doi: 10.1101/gad.212639.112, PMID: 23558772 PMC3639419

[137] Wang G-q Wang Y Xiong Y Chen X-C Ma M-l Cai R. Sirt1 AS lncRNA interacts with its mRNA to inhibit muscle formation by attenuating function of miR-34a. Sci Rep. (2016) 6:1865. doi: 10.1038/srep21865, PMID: 26902620 PMC4763196

[138] Cabianca Daphne S Casa V Bodega B Xynos A Ginelli E Tanaka Y. A Long ncRNA links copy number variation to a Polycomb/Trithorax epigenetic switch in FSHD muscular dystrophy. Cell. (2012) 149:819–31. doi: 10.1016/j.cell.2012.03.035 22541069 PMC3350859

[139] Jin JJ Lv W Xia P Xu ZY Zheng AD Wang XJ. Long noncoding RNA SYISL regulates myogenesis by interacting with polycomb repressive complex 2. Proceedings of the National Academy of Sciences (2018) 115:E9802–11. doi: 10.1073/pnas.1801471115, PMC6196504 30279181

[140] Song C Wang J Ma Y Yang Z Dong D Li H. Linc-smad7 promotes myoblast differentiation and muscle regeneration via sponging miR-125b. Epigenetics. (2018) 13:591–604. doi: 10.1080/15592294.2018.1481705, PMID: 29912619 PMC6140903

[141] Xiao T Liu L Li H Sun Y Luo H Li T. Long noncoding RNA ADINR regulates adipogenesis by transcriptionally activating C/EBPα. Stem Cell Rep. (2021) 16:1006–8. doi: 10.1016/j.stemcr.2021.03.024, PMID: 33852881 PMC8072130

[142] Zhu E Zhang J Li Y Yuan H Zhou J Wang B. Long noncoding RNA Plnc1 controls adipocyte differentiation by regulating peroxisome proliferator-activated receptor γ. FASEB J. (2019) 33:2396–408. doi: 10.1096/fj.201800739RRR, PMID: 30277818

[143] Sun Y Song Y Liu C Geng J. LncRNA NEAT1-MicroRNA-140 axis exacerbates nonalcoholic fatty liver through interrupting AMPK/SREBP-1 signaling. Biochem Biophys Res Commun. (2019) 516:584–90. doi: 10.1016/j.bbrc.2019.06.104, PMID: 31239155

[144] Nuermaimaiti N Liu J Liang X Jiao Y Zhang D Liu L. Effect of lncRNA HOXA11-AS1 on adipocyte differentiation in human adipose-derived stem cells. Biochem Biophys Res Commun. (2018) 495:1878–84. doi: 10.1016/j.bbrc.2017.12.006, PMID: 29217197

[145] Zhang X Xue C Lin J Ferguson JF Weiner A Liu W. Interrogation of nonconserved human adipose lincRNAs identifies a regulatory role of linc-ADAL in adipocyte metabolism. Sci Transl Med. (2018) 10:987. doi: 10.1126/scitranslmed.aar5987, PMID: 29925637 PMC6620026

[146] Huang Y Zheng Y Jin C Li X Jia L Li W. Long non-coding RNA H19 inhibits adipocyte differentiation of bone marrow mesenchymal stem cells through epigenetic modulation of histone deacetylases. Sci Rep. (2016) 6:897. doi: 10.1038/srep28897, PMID: 27349231 PMC4924093

[147] Cui X You L Li Y Zhu L Zhang F Xie K. A transcribed ultraconserved noncoding RNA, uc.417, serves as a negative regulator of brown adipose tissue thermogenesis. FASEB J. (2016) 30:4301–12. doi: 10.1096/fj.201600694R, PMID: 27655899

[148] Alvarez-Dominguez Juan R Bai Z Xu D Yuan B Lo Kinyui A Yoon Myeong J. De novo reconstruction of adipose tissue transcriptomes reveals Long non-coding RNA regulators of Brown adipocyte development. Cell Metab. (2015) 21:764–76. doi: 10.1016/j.cmet.2015.04.003, PMID: 25921091 PMC4429916

[149] Hotamisligil G Bai Z Chai X-r Yoon MJ Kim H-J Lo KA. Dynamic transcriptome changes during adipose tissue energy expenditure reveal critical roles for long noncoding RNA regulators. PLoS Biol. (2017) 15:76. doi: 10.1371/journal.pbio.2002176, PMID: 28763438 PMC5538645

[150] Zhao X-Y Li S Wang G-X Yu Q Lin JD. A Long noncoding RNA transcriptional regulatory circuit drives thermogenic adipocyte differentiation. Mol Cell. (2014) 55:372–82. doi: 10.1016/j.molcel.2014.06.004, PMID: 25002143 PMC4127104

[151] Agoulnik IU Liu S Xu R Gerin I Cawthorn WP MacDougald OA. SRA regulates Adipogenesis by modulating p38/JNK phosphorylation and stimulating insulin receptor gene expression and downstream signaling. PLoS One. (2014) 9:416. doi: 10.1371/journal.pone.0095416, PMID: 24743795 PMC3990642

[152] Huang Y Jin C Zheng Y Li X Zhang S Zhang Y. Knockdown of lncRNA MIR31HG inhibits adipocyte differentiation of human adipose-derived stem cells via histone modification of FABP4. Sci Rep. (2017) 7:131. doi: 10.1038/s41598-017-08131-6, PMID: 28808264 PMC5556051

[153] Firmin FF Oger F Gheeraert C Dubois-Chevalier J Vercoutter-Edouart A-S Alzaid F. The RBM14/CoAA-interacting, long intergenic non-coding RNA Paral1 regulates adipogenesis and coactivates the nuclear receptor PPARγ. Sci Rep. (2017) 7:14087. doi: 10.1038/s41598-017-14570-y, PMID: 29075020 PMC5658386

[154] Shang G Wang Y Xu Y Zhang S Sun X Guan H. Long non-coding RNA TCONS_00041960 enhances osteogenesis and inhibits adipogenesis of rat bone marrow mesenchymal stem cell by targeting miR-204-5p and miR-125a-3p. J Cell Physiol. (2018) 233:6041–51. doi: 10.1002/jcp.26424, PMID: 29319166 PMC5947671

[155] Chen Y Li K Zhang X Chen J Li M Liu L. The novel long noncoding RNA lncRNA-Adi regulates adipogenesis. Stem Cells Transl Med. (2020) 9:1053–67. doi: 10.1002/sctm.19-0438, PMID: 32356938 PMC7445023

[156] Ming Y Liu ZP. Overexpression of lncRNA-NEF regulates the miR-155/PTEN axis to inhibit adipogenesis and promote osteogenesis. Kaohsiung J Med Sci. (2021) 37:930–9. doi: 10.1002/kjm2.12423 34382731 PMC11896392

[157] Zhang T Liu H Mao R Yang H Zhang Y Zhang Y. The lncRNA RP11-142A22.4 promotes adipogenesis by sponging miR-587 to modulate Wnt5β expression. Cell Death Dis. (2020) 11:550. doi: 10.1038/s41419-020-2550-9, PMID: 32561739 PMC7305230

